# The longer-term impact of the COVID-19 pandemic on wellbeing and subjective cognitive functioning of older adults in Belgium

**DOI:** 10.1038/s41598-023-36718-9

**Published:** 2023-06-15

**Authors:** Sarah De Pue, Céline Gillebert, Eva Dierckx, Eva Van den Bussche

**Affiliations:** 1 Brain and Cognition, KU Leuven, Leuven, Belgium; 2 Personality and Psychopathology, Vrije Universiteit Brussel, Brussels, Belgium; 3 Psychiatric Hospital, Alexianen Zorggroep Tienen, Tienen, Belgium

**Keywords:** Geriatrics, Quality of life, Ageing, Human behaviour

## Abstract

In earlier survey research, we observed a severe impact of the first peak of the COVID-19 pandemic on the subjective wellbeing, sleep and activity of adults aged 65 years or older in Flanders, Belgium. The impact on subjective cognitive functioning, however, was limited. Since then, periods of lockdown and periods with less strict regulations alternated, but social distancing remained, especially for older adults. To study the longer-term impact of the pandemic on wellbeing and subjective cognitive functioning, we re-assessed the older adults from the first measurement moment (May–June 2020) in a second (June–July 2020) and third (December 2020) wave of the survey (*n* = 371, *M* = 72 years old, range 65–97 years old). Results indicated that wellbeing fluctuated with the severity of the pandemic. Results for self-reported cognitive functioning were mixed. While participants indicated a slightly better general subjective cognitive functioning at the end of the study, experienced problems with most cognitive subdomains significantly increased over time. The presence of depressive and anxiety symptoms were related to the longer-term impact of the pandemic on wellbeing and subjective cognitive functioning. Our study shows the long-lasting impact of the pandemic on the wellbeing and subjective cognitive functioning of older adults, without full recovery from the first wave.

## Introduction

In earlier research, we observed a severe impact of the first peak of the COVID-19 pandemic on the self-reported wellbeing of older adults aged 65 years or older in Flanders, Belgium^1^. The impact of the pandemic on subjective cognitive functioning on the other hand was rather limited. These findings were in line with other studies on the acute impact of the pandemic on older adults across the globe.

### The acute impact of the first peak of the pandemic on subjective wellbeing and cognitive functioning

As studies before the pandemic and during natural disasters already showed, social isolation and loneliness can negatively impact wellbeing and cognitive functioning of older adults^2–4^. As engagement in social activities and physical activity can be cognitively stimulating and help older adults cope with stress^5^, loss in social contact can lead to decreased wellbeing and cognitive functioning. In addition, worry and anxiety are known to impact working memory and thus cognitive performance^6,7^. Indeed, multiple studies during the first lockdown in March 2020 in other countries showed a decrease in *wellbeing* and an increase in reported depressive and anxiety symptoms and loneliness in older adults in comparison to the period before the pandemic^8–11^. Social support seemed to work as a protective buffer against these increases in depressive and anxiety symptoms^12^. Only a few studies looked at the impact of the early stages of the pandemic on *cognitive functioning* in older adults. Overall, they showed a decline in long-term memory^13^ associated with pandemic-related worries, and mild declines in subjective cognitive functioning^14^. From this, we can conclude that the first peak of the pandemic significantly decreased wellbeing and certain aspects of cognitive functioning to a limited extent. Importantly, the influence of vulnerability and protective factors such as depressive and anxiety symptoms and social network seemed to moderate these changes in wellbeing and cognitive functioning at the start of the pandemic.

Unfortunately, the COVID-19 pandemic did not end after its first peak. Periods of lockdown and less strict governmental regulations alternated. In Belgium, the first peak was followed by a period between May and July 2020, with less strict regulations, with shops reopening and social contact increasing again. However, by the end of October 2020, a new critical phase started, which resulted in a new lockdown from November 2020 to the end of April 2021^15^. Importantly, even in periods of less strict regulations, the recommendation of social distancing remained, especially for older adults. Since the COVID-19 pandemic took a large toll especially on older adults in terms of infection cases and deaths, older adults were considered as a vulnerable group during the pandemic and were the specific focus of governmental regulations. Because the COVID-19 pandemic lasted for an extended period, its impact on older adults was likely not limited to an immediate impact after the first peak. Therefore, studies on the sustained impact of the pandemic on wellbeing and cognitive functioning of this more vulnerable population and how this impact fluctuated throughout the phases of the pandemic are crucial.

### The longer-term impact of the pandemic on subjective wellbeing

Nation-wide surveys about the long-term impact of COVID-19 on mental health of the *general Belgian population* of 18 years and older on different time points during the pandemic by Sciensano^16,17^ showed fluctuations in depressive and anxiety symptoms. These symptoms seemed to increase in times of strict governmental regulations and high numbers of COVID-19 cases, but decreased in less severe pandemic times. Crucially, levels of anxiety and depressive symptoms always remained significantly higher than before the pandemic. Moreover, dissatisfaction with social support increased during lockdowns, and life satisfaction further decreased over time. Studies in other countries showed similar longer-term fluctuations in wellbeing depending on the severity of the pandemic (i.e. in terms of infection rates and subsequent governmental regulations)^18,19^ whereas others showed stable, low levels of mental health over the course of the pandemic^20^.

Specifically for *older adults*, longitudinal studies on wellbeing showed mixed findings. In line with observations for the general population, some studies observed that stress due to isolation and pandemic worry fluctuated with the severity of the pandemic^13,21^. Living with a partner and stronger relationships with family and friends played a protective role in these fluctuations over time^21^. Increased life purpose after the first lockdown was observed in older adults, associated with resilience and acceptance^22^. Contrarily, other studies indicated a steady further decline in wellbeing from the start of the pandemic to the second peak of the pandemic in November–December 2020^23,24^ or all-time low levels during different lockdown periods^25^.

### The longer-term impact of the pandemic on cognitive functioning

With regards to cognitive functioning, studies in the *general population* using online cognitive test batteries showed lower levels of processing speed and goal maintenance compared to before COVID-19 and during the first months of the pandemic. This decrease in cognitive functioning was related to pandemic worry^7^ and social isolation^26^.

Longitudinal studies on the impact of the pandemic on cognitive functioning of *older adults* are still very scarce. One study by Noguchi et al.^27^ in older adults found more subjective cognitive impairments as the pandemic continued in those participants that became or remained socially isolated. In a longitudinal study that started following older adults already before the pandemic, a steeper cognitive decline with time was observed since the pandemic, especially for memory and recall of word lists^28,29^. Moreover, higher anxiety symptoms in older adults were related to higher impairments in subjective cognitive functioning^30^.

So far, the sustained impact of the pandemic on wellbeing and cognitive functioning of older adults remains unclear and seems to be influenced by several protective and risk factors, such as depressive and anxiety symptoms, social network and resilience. We aimed to study the longer-term impact of the COVID-19 pandemic on the wellbeing and subjective cognitive functioning of older adults and how this longer-term impact fluctuated over the three measurement moments, in different phases of the pandemic. To achieve this, we extended our first measurement moment (i.e., T1, May–June 2020, right after the first lockdown in Belgium, on average 133 daily new COVID cases; see^1^) by assessing wellbeing and subjective cognitive functioning in the same group of older adults in a second wave (June–July 2020, T2, when COVID-19 cases and restrictions were low, on average 109 daily new cases) and a third wave (December 2020, T3, during the second lockdown in Belgium, on average 2127 daily new cases) of our survey study, reflecting different phases of the pandemic. Based on the scarce previous longitudinal studies, we expected declines in wellbeing and subjective cognitive functioning in more severe phases of the pandemic (i.e., T1 and T3 in our study), and improvements during periods with less strict governmental regulations (i.e., T2 in our study). Moreover, we studied the association with possible protective and vulnerability factors, which might be important based on the literature, namely cognitive failures, depressive and anxiety symptoms, social network, and resilience. The biopsychosocial model^31,32^ proposes that multiple biological, psychological (such as depression and anxiety) and social factors (such as perceived social support) can interact and influence subjective wellbeing, and are thus worth looking into. Theories such as the Socioemotional Selectivity theory^33^ particularly stress the buffering effect of social network against negative life-experiences in older adults, making this a crucial factor to take into consideration during the pandemic.

## Methods

### Participants

Participants who filled in the first part of our survey study (De Pue et al.,^1^) and agreed to be contacted again for the next waves by providing their contact details, were contacted again for the second and third wave of this study. Only participants who filled in at least 50% of the survey on T2 and T3 were included for analysis. This led to a total of 371 participants who were eligible for data analysis. As these data are based on a previous time point, no a priori power analyses were possible. Importantly, across the three waves, there was attrition. On T1, 640 older adults filled in the survey, of which 530 participants provided contact details for the next measurement moments. Of this group of participants, 453 older adults took part in the second wave of the study. On T3, 371 participants remained, who took part in all three measurement moments. There was thus a drop-out of 14.5% from the first to the second wave, and 30.0% from the first to the third wave. Supplementary Table 1 provides an overview of the main characteristics measured on T1 for the final sample of this manuscript versus those who dropped out after the first wave. We have complied with all relevant ethical regulations. All participants provided written informed consent. The second and third wave of this survey study were approved by the Social and Societal Ethics Committee (SMEC) from KU Leuven (G-2020–1987-R2(AMD)). All methods were performed in accordance with the relevant guidelines. For their participation, participants could win one of 16 gift certificates via a random draft on each measurement moment.

**Figure 1:**
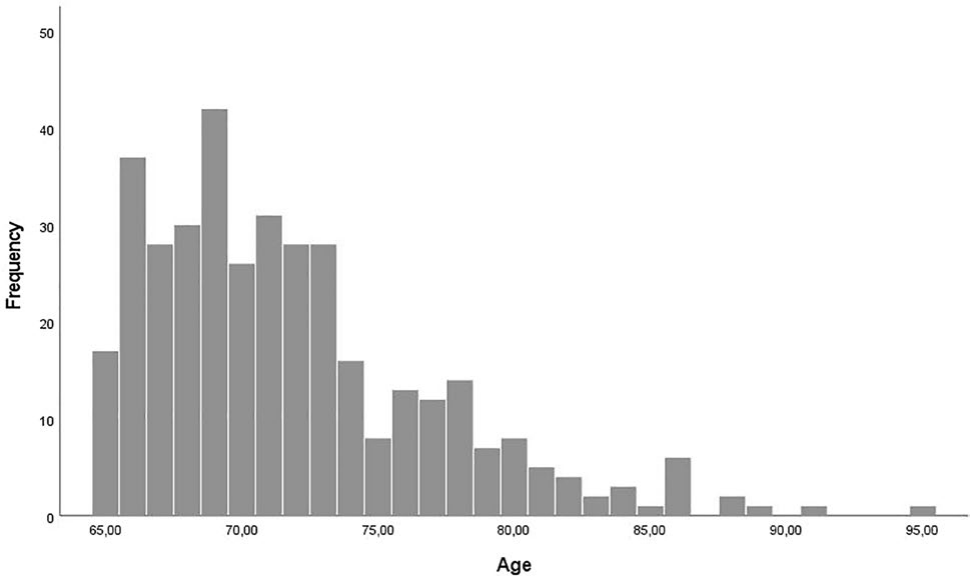
Frequency distribution of age.

Supplementary Table 1 contains the characteristics measured at T1 for the 371 participants who took part in all three measurement moments. Participants were on average 72 years old (*SD* = 5.30, range 65–95). A frequency distribution of age is depicted in Fig. 1. Around 46% of the participants were male. Most of the participants had the Belgian nationality and all participants lived across Flanders. Most participants lived in their own house, 7% lived in a nursing home or assisted living facility. The majority of the participants (58%) lived with one cohabitant, 11% lived with 2 or more cohabitants and 31% lived alone. Most participants (63%) were highly educated and had a university or high school degree. Most participants had a monthly individual net income between €1001–1500 (22%), €1501–2000 (35%) or €2001–2500 (22%). Almost all participants were retired and in good health. The percentage of participants contaminated with COVID-19 during T1, T2 and T3 was 4%, 4%, 3% respectively. Participants indicated that 15%, 21% and 26% of their family or friends had been contaminated with COVID-19 during T1, T2 and T3 respectively. On T2, 95% of the participants indicated they were willing to be vaccinated against COVID-19 if there would be a vaccine available. Supplementary Table 2 offers information on the number of contacts that participants had in different situations (e.g., inside, outside, telephone, internet). Participants had more contacts inside and outside with people during T2 compared to T1 and T3. The number of contacts through telephone and internet was rather stable across all moments. We note that all analyses in this manuscript have been rerun excluding participants who reported a COVID-19 infection on any of the three time points (*n* = 353), but as results remained highly similar, we only report the analyses including these participants.

### Material

In line with the first part of the survey study^1^, an online Qualtrics survey was used^34^. The survey addressed some general and demographic questions, and several questionnaires. Note that the current paper specifically focuses on wellbeing and subjective cognitive functioning and additionally on cognitive failures, depressive and anxiety symptoms, social network and resilience as potential protective and vulnerability factors. However, other variables were also assessed in the survey but are not reported or analyzed here (i.e., sleep, activity level, coping strategies assessed using the Cognitive Emotion Regulation Questionnaire-Short (CERQ-short)^35^). We refer the reader to the complete datafile and accompanying metadata which are openly shared on OSF for more information on the variables that were outside the scope of the current paper (https://osf.io/vfwus/). Table 1 displays an overview of which variables, included in the current study, were measured on which moment. Subjective wellbeing, subjective cognitive functioning, frequency of cognitive failures and depressive symptoms were assessed on each measurement moment. Subjective wellbeing and subjective cognitive functioning were also retrospectively assessed for the period before COVID-19 (Pre). Social network and resilience were only measured at T1 and anxiety symptoms only at T3. Details on the measures assessed at T1 (i.e., wellbeing, subjective cognitive functioning, depressive symptoms, social network and resilience), such as psychometric properties, can be found in De Pue et al.^1^, but we shortly summarize the most important information here.

**Table 1:** An overview of which outcome and protective or vulnerability factor was assessed on which measurement moment.

		Pre	T1 May 19–June 22 2020	T2 June 16–July 16 2020	T3 December 9–30 2020
*Changes in subjective cognitive functioning*	General subjective cognitive functioning	X	Only in %	X	X
	Problems with cognitive subdomains	–	X	X	X
Changes in wellbeing: PWI-A		X	X	X	X
Cognitive failures: CFQ		–	X	X	X
Depressive symptoms: GDS-15		–	X	X	X
Social network: LSNS-6		–	X	–	–
Resilience: BRS		–	X	–	–
Anxiety symptoms: HADS		–	–	–	X

The Pre-measurement moment was assessed retrospectively.

*PWI-A* Personal Wellbeing Index-Adults, *CFQ* Cognitive Failures Questionnaire, *GDS-15* Geriatric Depression Scale-15, *LSNS-6* Lubben Social Network Scale-6, *BRS* Brief Resilience Scale, *HADS* the anxiety items of the Hospital Anxiety and Depressive Symptoms questionnaire.

On **T1**, we assessed age, gender, country of residence, nationality, postal code, living situation, educational level, current and previous work situation, monthly individual net income, age-related diseases and whether the participant and/or any of their close relatives or friends had been infected with COVID-19.

#### Changes in subjective cognitive functioning (Pre, T1, T2 and T3)

On **T1**, we assessed if *general subjective cognitive functioning* had changed during the COVID-19 period using a 3-point scale: Yes, it has decreased (= 1); No, it has not changed (= 2); Yes, it has improved (= 3). In order to be able to compare subjective cognitive functioning between the different measurement moments in a more fine-grained way, this format was changed on **T2 and T3**. Now, participants had to evaluate their cognitive functioning on a scale from 0 (very bad) to 10 (very good). This was assessed on T2 for the past month and also retrospectively for the period before COVID-19 (**Pre**), and on T3 for the past month. In addition, on **T1, T2 and T3**, participants indicated the frequency of *problems with certain cognitive subdomains* (i.e., problems to remember things, to concentrate on something, to do two things at the same time, to recall things and forgetfulness) since the past month, on a 5-point scale with labels “a lot more than before” (= 1), “more than before” (= 2), “not more or less than before” (= 3), “less than before” (= 4), “a lot less than before” (= 5). The internal consistency of these subjective cognitive change questions was high with Cronbach’s *α* = 0.87, *α* = 0.84 and *α* = 0.86 on T1, T2 and T3 respectively.

#### Changes in wellbeing (Pre, T1, T2 and T3)

On every measurement moment as well as retrospectively before the pandemic, subjective wellbeing was assessed using the Dutch version of the *Personal Wellbeing Index-Adults* (PWI-A;^36,37^), which measures life satisfaction in different domains (i.e., general life satisfaction, standard of living, health, achieving in life, relationships, safety, community connectedness and future security). The eight items, i.e. one item for each subdomain, are scored on an 11-point scale ranging from “no satisfaction at all” (= 0) to “completely satisfied” (= 10), and converted to a 0–100 scale, with higher scores indicating more satisfaction. An index for general subjective wellbeing is calculated by summing the seven domain items (excluding the general life satisfaction item). The internal consistency in the current study was high for the PWI-A total score (i.e., 7 items) with Cronbach’s *α* = 0.89, *α* = 0.89, *α* = 0.90 and *α* = 0.89 on Pre, T1, T2 and T3 respectively.

#### Frequency of cognitive failures (T1, T2 and T3)

The Dutch version of the *Cognitive Failures Questionnaire* (CFQ;^38,39^) was used to assess frequency of cognitive failures on T1, T2 and T3. Participants answered 25 items assessing self-reported frequency of failures in several cognitive domains during the past month, rated on a 5-point scale ranging from “very often” (= 4) to “never” (= 0). We also added a response option “not applicable”. Items included for example “Do you read something and find you haven’t been thinking about it and must read it again?” or “Do you find you forget people’s names?”. A total score across all items, varying between 0 and 100, provides a measure of the general susceptibility to cognitive failures with a higher score indicating a higher susceptibility. The “not applicable” scores were not included to calculate the total score. For participants who indicated “not applicable” on more than 50% of the items (*n* = 7 on T1, *n* = 4 on T2 and *n* = 2 on T3), no CFQ total score was computed. The internal consistency of the CFQ was high with Cronbach’s *α* = 0.91, *α* = 0.92 and *α* = 0.92 on T1, T2 and T3 respectively.

#### Depressive symptoms (T1, T2 and T3)

Depressive symptoms were assessed with the *Geriatric Depression Scale-15* (GDS-15;^40,41^) on every measurement moment. Participants answered 15 Yes/No items about the past month (e.g., “Do you feel that your life is empty?” or “Are you afraid that something bad is going to happen to you?”). Items are summed to a total score between 0 and 15, with higher scores indicating more depressive symptoms. The internal consistency of the GDS-15 was good with Cronbach’s *α* = 0.78, *α* = 0.80 and *α* = 0.80 on T1, T2 and T3 respectively.

#### Social network (T1)

The *Lubben Social Network Scale-6* (LSNS-6;^42^), was used to assess social network, with six items evaluating family ties and non-family ties. These items are scored on a 6-point scale where participants indicate the number of ties (i.e., 0, 1, 2, 3 or 4, 5 to 8, 9 or more). A sum score is calculated, ranging between 0 and 30, with a higher score indicating more social engagement. Next to the LSNS-6, we also asked participants how many contacts they had (not taking into account cohabitants) during the past week in real life outside, in real life inside, by telephone and via the internet (e.g., skype, whatsapp), using the same response scale of the LSNS-6. The internal consistency of the LSNS-6 on T1 was high with Cronbach’s *α* = 0.81.

#### Resilience (T1)

Resilience was assessed using the Dutch version of the *Brief Resilience Scale* (BRS;^43,44^). The six items are scored on a 5-point scale ranging from “strongly disagree” to “strongly agree”. After reversing items 2, 4 and 6, a mean score is calculated which ranges between 1 and 5, with a higher score indicating more resilience. The internal consistency of the BRS on T1 was high with Cronbach’s *α* = 0.80.

#### Anxiety (T3)

To measure reported anxiety symptoms, the items assessing anxiety from the Dutch version of the Hospital Anxiety Depression Scale (HADS^45,46^) were used. These 7 items are scored on a 4-point scale from 0 (not at all) to 3 (almost always/certainly). A sum score of the 7 items is calculated, ranging from 0 to 21, with a higher score indicating more anxiety symptoms. The psychometric properties of the anxiety items of the HADS were reported as good in Dutch healthy older adults^46^. The internal consistency of the HADS on T3 was good with Cronbach’s alpha = 0.87.

### Procedure

Participants of T1 (May–June 2020) were contacted to participate on T2 (June–July 2020), approximately one month after participating on T1 (*M* = 26 days, *SD* = 3.09, range 21–40 days). After giving their consent, they had to fill in their birth year, age, postal code and indicate if they or their family or friends were infected with COVID. Next, they completed the CFQ and general subjective cognitive questions focusing on the past month and retrospectively on the period before COVID-19. After that, the GDS-15, questions about activity and sleep, the PWI-A and the questions about social contacts were completed. At the end, participants could provide their contact details to indicate that we could contact them to participate on T3, and/or to win a gift certificate. Participants of T2 were again contacted to participate on T3 (December 2020), approximately six months after T2 (*M* = 6 months, *SD* = 0.31, range 5–7 months). T3 was identical to T2, except for the addition of the HADS and the CERQ-short. The median completion time of the survey was 20, 26 and 35 min on T1, T2 and T3 respectively (note that this time was longer for T2 and T3 because after our survey, participants also took part in other short assessments on T2 and T3 unrelated to the current study). Responses of an individual participant on the three measurement moments were merged using a unique code participants had to fill in on each moment, formed by the first two letters of their name, their birth year and the first two letters of the city they live in.

### Statistical analyses

First, Pearson correlations between the different outcome measures (i.e. general subjective cognitive functioning and wellbeing ratings), age and the protective and risk factors used in this study (i.e. cognitive failures, depressive symptoms, social network, resilience and anxiety symptoms) were reported. Based on Cohen^48^, correlations were considered as small, medium or strong when the correlation coefficient was below 0.30, between 0.30 and 0.50 and above 0.50. Moreover, mean subjective cognitive functioning ratings and wellbeing scores for the different measurement moments, as well as mean scores for the protective and vulnerability factors included in the current study were reported. Supplementary Tables 3 and 4 show a comparison between the mean scores of the main study variables for participants that are included in the study versus those who dropped out before the third wave.

Next, the effect of the different phases of the pandemic on subjective cognitive functioning and wellbeing was studied. Repeated measures ANOVAs were conducted with time as within-subject factor and self-reported ratings of cognitive functioning on a scale from 0 to 10 (measured Pre, T2 and T3), general subjective wellbeing scores and the scores on the different wellbeing subdomains (i.e., the PWI-A scores; measured Pre, T1, T2 and T3) as outcome variables. Post-hoc paired samples *t*-tests with Bonferroni correction were used to compare scores on the outcome measures between different measurement moments. In addition, the percentages of participants reporting more problems on the different cognitive subdomains since the past month for each measurement moment were reported (measured T1, T2 and T3). Moreover, dichotomous variables were created for each subdomain and measurement moment, with 0 = participants who reported less or an equal amount of cognitive problems since the past month, and 1 = participants who reported more problems on that cognitive domain since the past month. Cochrans’ Q tests were used for each cognitive subdomain, with the dichotomous variables of that subdomain on each measurement moment as dependent variables. When significant, Bonferroni corrected McNemar’s tests were used as post-hoc tests to compare proportions of participants reporting more problems on that cognitive subdomain between the different measurement moments. Effect sizes of significant post-hoc McNemar’s tests were reported as odds ratio’s, calculated by b/c with b = number of participants reporting less or an equal amount of problems on that cognitive subdomain since the past month on T1 or T2 but more problems on T2 or T3 respectively, and c = number of participants reporting more problems on that cognitive subdomain on T1 or T2 but less or an equal amount of problems on T2 or T3 respectively^47^.

Third, to study the association between protective and vulnerability factors and these observed changes in subjective cognitive functioning and wellbeing, repeated measures ANOVAs were used with time as within-subject factor and adding between-subject factors and covariates. In line with our previous study focused on T1^1^, gender, living assisted or not and living alone or not were added as between-subject factors, and age and monthly net income were added as covariates. Furthermore, frequency of cognitive failures, depressive symptoms, social network, resilience and anxiety symptoms were added as covariates, as they were potential protective and vulnerability factors. For frequency of cognitive failures, depressive symptoms, social network and resilience, the scores of T1 were used, and for anxiety, the scores of T3 were used (as this was only measured on T3). Note that health status, educational attainment and residence could not be included in the analyses given the imbalanced number of participants in the separate conditions of these variables. To visualize significant interactions between time and a continuous covariate, a binning procedure was applied to the covariate, dividing the variable into four bins. Using SPSS version 28, the respective covariate was binned using the Visual binning function, generating four bins with equal percentiles of cases based on the data (i.e., each bin containing around 25% of the cases, i.e. a quartile split). These four bins represented participants with the lowest (bin 1), second lowest (bin 2), second highest (bin 3) and highest (bin 4) scores on that respective protective or risk factor. To further interpret significant interactions, a difference score was calculated by subtracting the score on the outcome variable (i.e., cognitive functioning or wellbeing) before the pandemic, assessed retrospectively, from the score of that outcome variable on T3 (T3-Pre). Hence, negative difference scores indicated a decrease in that outcome variable since before the pandemic. A one-way ANOVA with post-hoc independent samples *t*-tests with Bonferroni correction was then conducted to study if the difference score differed significantly between the different bins of the protective and vulnerability factors. In case this ANOVA with difference scores T3-Pre could not explain the significant interaction in the repeated measures ANOVA, ANOVAs with the other difference scores (i.e., T2-Pre, T1-Pre, T3-T2, T3-T1 and T2-T1) were conducted.

Partial $\eta^{2}$ and Cohen’s *d* were reported as effect sizes. We considered effect sizes as small, medium or strong when $\eta_{p}^{2}$ was around 0.01, 0.06 and 0.14, and when the Cohen’s *d* was around 0.20, 0.50 and 0.80, respectively (based on Cohen^48^).

## Results

Pearson correlations between the ratings of general subjective cognitive functioning and wellbeing on T1, T2 and T3 and the protective and risk variables are shown in Table 2.

**Table 2:** Pearson correlations between the outcomes and the protective and vulnerability factors in this study.

	Age	CFQ	GDS-15	LSNS-6	BRS	HADS	PWI-A Pre	PWI-A T1	PWI-A T2	PWI-A T3	Cognition Pre	Cognition T2	Cognition T3
Age	–	− .16**	− .054	− .048	.11*	− .16*	.007	.054	.015	.034	.005	.032	− .010
CFQ		–	.27***	− .049	− .32***	− .31***	− .32***	− .33***	− .34***	− .27***	− .49***	− .56***	− .45***
GDS-15			–	− .31***	− .45***	.56***	− .38***	− .64***	− .57***	− .60***	− .15**	− .39***	− .44***
LSNS-6				–	.13*	− .23***	.34***	.37***	.32***	.37***	− .003	.12*	.24***
BRS					–	− .49***	.44***	.46***	.49***	.48***	.25***	.33***	.33***
HADS						–	− .33***	− .52***	− .54***	− .67***	− .18***	− .37***	− .48***
PWI-A Pre							–	.73***	.68***	.64***	.38***	.42***	.43***
PWI-A T1								–	.77***	.76***	.31***	.47***	.50***
PWI-A T2									–	.78***	.36***	.50***	.52***
PWI-A T3										–	.28***	.42***	.57***
Cognition Pre											–	.82***	.47***
Cognition T2												–	.61***
Cognition T3													–

*CFQ* Cognitive Failures Questionnaire total score, *GDS-15* Geriatric Depression Scale-15 total score, *LSNS-6* Lubben Social Network Scale-6 total score, *BRS* Brief Resilience Scale mean score, *HADS* Hospital Anxiety and Depressive symptoms sum score on the anxiety items, *PWI-A* Personal Wellbeing Index-Adults total score retrospectively assessed for the pre-pandemic period, T1, T2 and T3, Cognition = the subjective cognitive function question retrospectively assessed for the pre-pandemic period, T2 and T3.

***p < .001, **p < .010, *p < .050.

### Changes in subjective cognitive functioning and wellbeing over the course of the pandemic

**Figure 2:**
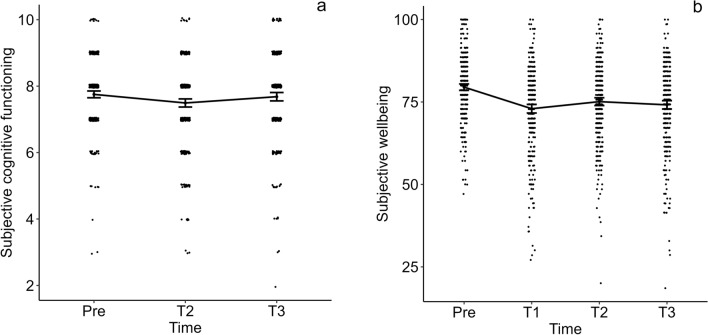
Changes in subjective cognitive functioning (**a**) and general wellbeing (**b**) over the course of the pandemic.

Table 3 and Fig. 2 show the reported subjective cognitive functioning ratings and wellbeing scores for the different measurement moments.

**Table 3:** Means (*SD*s) of reported subjective cognitive functioning and wellbeing on the different measurement moments, and percentages of participants reporting more problems with specific subdomains of subjective cognitive functioning since the past month.

		Pre	T1	T2	T3
	*n*	Mean (*SD*)	Mean (*SD*)	Mean (*SD*)	Mean (*SD*)
*Subjective cognitive functioning*					
General cognitive functioning rating	371	7.75 (0.99)	–	7.49 (1.20)	7.68 (1.24)
Problems with remembering	371	–	7%	7%	16%
Problems with concentrating	371	–	12%	13%	17%
Problems with doing two things at the same time	371	–	5%	9%	15%
Problems with recalling	371	–	9%	11%	26%
Problems with forgetfulness	371	–	8%	10%	21%
*Wellbeing (PWI-A)*					
General subjective wellbeing	371	79.50 (9.50)	72.96 (12.78)	75.11 (11.58)	74.14 (12.39)
General life satisfaction	371	79.84 (11.27)	71.02 (17.07)	74.47 (13.73)	73.29 (14.87)
Standard of living	371	81.37 (11.22)	78.89 (13.46)	80.46 (11.56)	80.81 (12.14)
Health	371	77.79 (12.28)	75.15 (14.75)	76.71 (14.39)	74.72 (15.99)
Achieving in life	371	79.92 (12.25)	76.42 (15.19)	78.30 (12.08)	77.20 (14.28)
Relationships	371	79.76 (14.03)	72.94 (19.19)	75.50 (15.73)	74.88 (18.13)
Safety	371	81.78 (10.00)	71.94 (16.34)	74.37 (15.13)	74.39 (15.97)
Community connectedness	371	78.01 (13.19)	68.01 (18.00)	69.60 (17.71)	67.76 (18.32)
Future security	371	77.87 (11.67)	67.39 (16.73)	70.81 (15.51)	69.19 (16.79)

*PWI-A* Personal Wellbeing Index-Adults total score.

#### Changes in subjective cognitive functioning over time

On T1, participants simply indicated whether their cognitive functioning has changed since before COVID-19 and 7% indicated that their cognitive functioning had worsened. On all measurement moments, participants indicated whether they experienced more, equal or less problems with several subdomains of cognitive functioning since the lockdown (on T1) or since past month (on T2 and T3). Table 4 contains the test statistics of all analyses and accompanying Bonferroni corrected post-hoc tests to compare the proportion of participants reporting more problems with these cognitive subdomains over time. To summarize, the percentage of participants reporting more problems with remembering, concentrating, doing two things at the same time, recalling and forgetfulness since the past month remained rather stable from the first to the second measurement moment (T1 to T2). Crucially, this percentage of participants reporting more problems with cognitive subdomains significantly increased towards the third measurement moment for all subdomains (see Tables 3 and 4), especially for problems with recalling.

**Table 4:** Statistical results of the Cochran’s Q tests with the dichotomous cognitive subdomain variable, reflecting if participants have more problems with that cognitive subdomain or not, on each measurement moment as dependent variable, and the accompanying Bonferroni corrected post-hoc McNemar’s tests.

Cognitive subdomain	Cochran’s Q test statistics	Post-hoc comparisons (Bonferroni correction *α* = .017)
Remembering	*χ*^2^(2) = 31.32, *p* < .001	T1 versus T2: *p* = .85
		T1 versus T3: *χ*^2^(1) = 17.36, *p* < .001, OR = 3.50
		T2 versus T3: *χ*^2^(1) = 18.35, *p* < .001, OR = 3.54
Concentration	*χ*^2^(2) = 9.44, *p* = .009	T1 versus T2: *p* = .64
		T1 versus T3: *χ*^2^(1) = 6.56, *p* = .010, OR = 2.05
		T2 versus T3: *p* = .034
Doing two things at the same time	*χ*^2^(2) = 28.91, *p* < .001	T1 versus T2* p* = .035
		T1 versus T3: *χ*^2^(1) = 22.92, *p* < .001, OR = 4.25
		T2 versus T3: *χ*^2^(1) = 9.44, *p* = .002, OR = 2.39
Recalling	*χ*^2^(2) = 68.13, *p* < .001	T1 versus T2: *p* = .53
		T1 versus T3: *χ*^2^(1) = 44.18, *p* < .001, OR = 4.83
		T2 versus T3: *χ*^2^(1) = 36.92, *p* < .001, OR = 3.41
Forgetfulness	*χ*^2^(2) = 40.66, *p* < .001	T1 versus T2: *p* = .54
		T1 versus T3: *χ*^2^(1) = 28.93, *p* < .001, OR = 6.25
		T2 versus T3: *χ*^2^(1) = 21.33, *p* < .001, OR = 4.87

A repeated measures ANOVA with time as within-subject factor on the self-reported ratings of cognitive functioning on a scale from 0 to 10 (see Fig. 2a) showed a significant main effect of time (*F*(2,369) = 29.07, *p* < 0.001, $\eta_{p}^{2}$ = 0.14). Post-hoc paired samples *t*-tests with Bonferroni correction (*α* = 0.017) showed that self-rated cognitive functioning was significantly lower on T2 (*M* = 7.49) compared to the period before COVID-19 (*M* = 7.75) which was measured retrospectively, *t*(370) = 7.33, *p* < 0.001, *d* = 0.38. Self-rated cognitive functioning on T3 (*M* = 7.68) was significantly higher than on T2 (*M* = 7.49), *t*(370) = -3.38, *p* < 0.001, *d* = -0.18. The difference in subjective cognitive functioning before COVID and on T3 was not significant (*p* = 0.25).

#### Changes in subjective wellbeing over time

Table 5 provides a full description of all analyses and accompanying post-hoc tests with Bonferroni correction to follow-up significant effects for changes in the different domains of wellbeing.

**Table 5:** Statistical results of the repeated measures ANOVAs with the PWI-A subdomains as outcome and time as within-subject factor, and the accompanying Bonferroni corrected post-hoc tests.

PWI-A subdomain	Main effect of time	Post-hoc comparisons (Bonferroni correction *α* = .008)
General subjective wellbeing	*F*(3,368) = 73.34*, p* < .001*,* $\eta_{p}^{2}$ = .37	Pre versus T1: *t*(370) = 14.49,* p* < .001,* d* = 0.75
		Pre versus T2: *t*(370) = 9.75,* p* < .001,* d* = 0.51
		Pre versus T3: *t*(370) = 10.67,* p* < .001,* d* = 0.55
		T1 versus T2: *t*(370) = − 4.95,* p* < .001,* d* = − 0.26
		T1 versus T3: *p* = .010
		T2 versus T3: *p* = .020
General life satisfaction	*F*(3,368) = 56.42, *p* < .001, $\eta_{p}^{2}$ = .32	Pre versus T1: *t*(370) = 12.20, *p* < .001, *d* = 0.63
		Pre versus T2: *t*(370) = 8.18, *p* < .001, *d* = 0.43
		Pre versus T3: *t*(370) = 9.78, *p* < .001, *d* = 0.51
		T1 versus T2: *t*(370) = − 5.21, *p* < .001, *d* = − 0.27
		T1 versus T3: *t*(370) = − 3.15, *p* = .002, *d* = − 0.16
		T2 versus T3: *p* = .076
Standard of living	*F*(3,368) = 11.34, *p* < .001, $\eta_{p}^{2}$ = .085	Pre versus T1: *t*(370) = 5.66, *p* < .001, *d* = 0.29
		Pre versus T2: *p* = .077
		Pre versus T3: *p* = .32
		T1 versus T2: *t*(370) = − 3.12, *p* = .002, *d* = − 0.16
		T1 versus T3: *t*(370) = − 3.41, *p* < .001, *d* = − 0.18
		T2 versus T3: *p* = .47
Health	*F*(3,368) = 14.56, *p* < .001, $\eta_{p}^{2}$ = .11	Pre versus T1: *t*(370) = 5.61, *p* < .001, *d* = 0.29
		Pre versus T2: *p* = .089
		Pre versus T3: *t*(370) = 4.28, *p* < .001, *d* = 0.22
		T1 versus T2: *t*(370) = − 2.68, *p* = .008, *d* = − 0.14
		T1 versus T3: *p* = .54
		T2 versus T3: *t*(370) = 3.08, *p* = .002, *d* = 0.16
Achieving in life	*F*(3,368) = 14.71, *p* < .001, $\eta_{p}^{2}$ = .11	Pre versus T1: *t*(370) = 6.33, *p* < .001, *d* = 0.33
		Pre versus T2: *t*(370) = 2.75, *p* = .006, *d* = 0.14
		Pre versus T3: *t*(370) = 4.41, *p* < .001, *d* = 0.23
		T1 versus T2: *t*(370) = − 3.11, *p* = .002, *d* = − 0.16
		T1 versus T3: *p* = .21
		T2 versus T3: *p* = .050
Relationships	*F*(3,368) = 34.97, *p* < .001, $\eta_{p}^{2}$ = .22	Pre versus T1: *t*(370) = 9.63, *p* < .001, *d* = 0.50
		Pre versus T2: *t*(370) = 6.31, *p* < .001, *d* = 0.33
		Pre versus T3: *t*(370) = 6.30, *p* < .001, *d* = 0.33
		T1 versus T2: *t*(370) = − 3.24, *p* = .001, *d* = − 0.17
		T1 versus T3: *p* = .020
		T2 versus T3: *p* = .39
Safety	*F*(3,368) = 60.88, *p* < .001, $\eta_{p}^{2}$ = .33	Pre versus T1: *t*(370) = 12.97, *p* < .001, *d* = 0.67
		Pre versus T2: *t*(370) = 9.89, *p* < .001, *d* = 0.51
		Pre versus T3: *t*(370) = 9.30, *p* < .001, *d* = 0.48
		T1 versus T2: *t*(370) = − 3.30, *p* = .001, *d* = − 0.17
		T1 versus T3: *t*(370) = − 3.22, *p* = .001, *d* = − 0.17
		T2 versus T3: *p* = .97
Community connectedness	*F*(3,368) = 58.59, *p* < .001, $\eta_{p}^{2}$ = .32	Pre versus T1: *t*(370) = 11.74, *p* < .001, *d* = 0.61
		Pre versus T2: *t*(370) = 9.77, *p* < .001, *d* = 0.51
		Pre versus T3: *t*(370) = 11.22, *p* < .001, *d* = 0.58
		T1 versus T2: *p* = .046
		T1 versus T3: *p* = .78
		T2 versus T3: *p* = .032
Future security	*F*(3,368) = 72.54, *p* < .001, $\eta_{p}^{2}$ = .37	Pre versus T1: *t*(370) = 14.17, *p* < .001, *d* = 0.74
		Pre versus T2: *t*(370) = 9.81, *p* < .001, *d* = 0.51
		Pre versus T3: *t*(370) = 10.40, *p* < .001, *d* = 0.54
		T1 versus T2: *t*(370) = − 4.67, *p* < .001, *d* = − 0.24
		T1 versus T3: *p* = .024
		T2 versus T3: *p* = .022

##### Changes in general subjective wellbeing

A repeated measures ANOVA with time as within-subjects factor on the general subjective wellbeing scores measured on a scale from 0 to 100 (see Fig. 2b) showed a main effect of time (*F*(3,368) = 73.34, *p* < 0.001, $\eta_{p}^{2}$ = 0.37). Post-hoc paired samples *t*-tests with Bonferroni correction (*α* = 0.008) showed that general subjective wellbeing was significantly higher in the period before COVID-19 (*M* = 79.50), retrospectively measured, compared to T1 (*M* = 72.96, *t*(370) = 14.49, *p* < 0.001, *d* = 0.75), T2 (*M* = 75.11, *t*(370) = 9.75, *p* < 0.001, *d* = 0.51) and T3 (*M* = 74.14, *t*(370) = 10.67, *p* < 0.001, *d* = 0.55). General subjective wellbeing on T1 (*M* = 72.96) was significantly lower than on T2 (*M* = 75.11, *t*(370) = -4.95, *p* < 0.001, *d* = -0.26). General subjective wellbeing did not differ significantly between T1 and T3 and between T2 and T3 (*p* ≥ 0.010).

##### Changes in the different subdomains of subjective wellbeing

Repeated measures ANOVAs with time as within-subjects factor and each of the PWI-A subdomains (i.e., general life satisfaction, satisfaction with standard of living, health, achieving in life, relationships, safety, community connectedness and future security) as outcome were conducted. As shown in Table 5, generally, ratings of wellbeing for the subdomains were significantly higher before COVID-19, measured retrospectively, compared to all three measurement moments. Moreover, ratings on T1 were significantly lower than on T2. Ratings on T1 versus T3 and T2 versus T3 did not differ significantly from each other for most wellbeing subdomains. Mean ratings for each subdomain of wellbeing for the different measurement moments are included in Table 3.

To shortly summarize, after the negative impact of the first peak of the pandemic, subjective wellbeing fluctuated over the three measurement moments with fluctuating severity of the pandemic. Results for subjective cognitive functioning were mixed. While participants indicated a slightly better general subjective cognitive functioning at the end of the study, similar to the level of subjective cognitive functioning before the pandemic, problems in different subdomains of cognitive functioning significantly increased towards the last measurement moment.

### The association between protective and vulnerability factors and the changes in subjective cognitive functioning and wellbeing

Table 6 displays the mean scores for the protective and vulnerability factors included in the current study.

**Table 6:** Mean scores and standard deviations for the protective and vulnerability factors included in the current study.

	Range	T1		T2		T3	
		*n*	Mean (*SD*)	*n*	Mean (*SD*)	*n*	Mean (*SD*)
Cognitive failures (CFQ)	0–100	364	22.61 (11.25)	367	24.50 (11.40)	369	26.85 (11.86)
Depressive symptoms (GDS-15)	0–15	371	2.60 (2.66)	371	2.59 (2.80)	371	2.77 (2.80)
Social network (LSNS-6)	0–30	371	17.54 (5.22)	–	–	–	–
Resilience (BRS)	1–5	371	3.40 (0.65)	–	–	–	–
Anxiety symptoms (HADS)	0–21	–	–	–	–	371	4.22 (3.65)

*CFQ* Cognitive Failures Questionnaire total score, *GDS-15* Geriatric Depression Scale-15 total score, *LSNS-6* Lubben Social Network Scale-6 total score, *BRS* Brief Resilience Scale mean score, *HADS* Hospital Anxiety and Depressive symptoms sum score on the anxiety items.

#### Protective and risk factors and changes in subjective cognitive functioning.

A repeated measures ANOVA was conducted with subjective cognitive functioning as outcome, time as within-subject factor, gender, living assisted or not and living alone or not as between-subject factors and age, monthly net income, frequency of cognitive failures, depressive symptoms, social network, resilience and anxiety symptoms as covariates. This analysis showed significant main effects of frequency of cognitive failures (*F*(1, 349) = 116.70, *p* < 0.001, $\eta_{p}^{2}$ = 0.25), depressive symptoms (*F*(1, 349) = 6.79, *p* = 0.010, $\eta_{p}^{2}$ = 0.020) and anxiety symptoms (*F*(1, 349) = 7.01, *p* = 0.008, $\eta_{p}^{2}$ = 0.020). As can be seen on Supplementary Figure 1, participants with a higher frequency of cognitive failures (panel a), higher depressive (panel b) or anxiety symptoms (panel d) show overall lower ratings of subjective cognitive functioning. This pattern is the most prominent for those participants with the highest cognitive failures and depressive symptoms (i.e., bin 4). Moreover, significant interaction effects between time and frequency of cognitive failures (*F*(2, 348) = 5.03, *p* = 0.007, $\eta_{p}^{2}$ = 0.028), time and depressive symptoms (*F*(2, 348) = 19.35, *p* < 0.001, $\eta_{p}^{2}$ = 0.10), time and social network (*F*(2, 348) = 4.25, *p* = 0.015, $\eta_{p}^{2}$ = 0.024) and time and anxiety symptoms (*F*(2, 348) = 10.98, *p* < 0.001, $\eta_{p}^{2}$ = 0.059), were present. None of the other main effects and interactions were significant (*p* ≥ 0.075). To interpret the four significant interactions, we first used one-way ANOVAs to compare the T3-Pre difference score for subjective cognitive functioning (i.e., subjective cognitive functioning measured on T3—before the pandemic measured retrospectively) between the different bins of the covariate. This allowed us to study whether changes in subjective cognitive functioning from pre-COVID to T3 were related to varying levels of the covariate. Supplementary Table 5 contains the mean difference scores for subjective cognitive functioning for each bin depending on the protective or vulnerability factor. If this analysis was not sufficient (i.e., the ANOVA was not significant), we explored the difference score for cognitive functioning between pre-COVID and T2 and between T2 and T3 in order to capture the interaction.

##### Interaction between time and frequency of cognitive failures

A one-way ANOVA with M3-Pre difference score for subjective cognitive functioning as dependent variable and cognitive failures bins (i.e. based on the CFQ score) as between-subject factor was not significant, *F*(3,360) = 1.26, *p* = 0.29, $\eta_{p}^{2}$ = 0.010. Therefore, to further explore the interaction, one-way ANOVAs with the T2-Pre (*F*(3,360) = 5.95, *p* < 0.001, $\eta_{p}^{2}$ = 0.047) and the T3-T2 (*F*(3,360) = 0.91, *p* = 0.44, $\eta_{p}^{2}$ = 0.008) difference score as dependent variable and cognitive failures bins as between-subject factor were conducted as well. Post-hoc independent samples *t*-tests with Bonferroni correction (*α* = 0.008) for the significant ANOVA with difference score T2-Pre showed that participants with the highest frequency of cognitive failures (bin 4, *M* = -0.51) had a significantly steeper decrease in subjective cognitive functioning from before the pandemic, measured retrospectively, to T2 compared to participants with the lowest (bin 1, *M* = -0.16, *t*(132.94) = 3.23,* p* = 0.002, *d* = 0.50) and second lowest frequency of cognitive failures (bin 2, *M* = -0.12, *t*(137.63) = 3.58,* p* < 0.001, *d* = 0.55) . This is visually presented in Supplementary Figure 1a. All other comparisons were not significant (*p* ≥ 0.057).

##### Interaction between time and depressive symptoms

The one-way ANOVA with T3-Pre difference score for subjective cognitive functioning as dependent variable and depressive symptoms bins (i.e. based on the GDS-15 score) as between-subject factor was significant, *F*(3,367) = 16.31, *p* < 0.001, $\eta_{p}^{2}$ = 0.12. Post-hoc independent samples *t*-tests with Bonferroni correction (*α* = 0.008) showed that participants with the highest depressive symptoms (bin 4, *M* = -0.78) differed significantly in subjective cognitive function difference score from participants with the lowest depressive symptoms (bin 1, *M* = 0.055, *t*(121.57) = 4.70,* p* < 0.001, *d* = 0.72), participants with the second lowest depressive symptoms (bin 2, *M* = 0.28, *t*(153.78) = 5.25, *p* < 0.001, *d* = 0.81) and participants with the second highest depressive symptoms, (bin 3, *M* = 0.24, *t*(125.73) = 5.25, *p* < 0.001, *d* = 0.79). Whereas participants with the highest depressive symptoms showed a negative difference score, indicating a decrease in subjective cognitive functioning over time, all other bins showed slightly positive difference scores, or increases in cognitive functioning over time. This is visually presented in Supplementary Figure 1b. All other comparisons were not significant (*p* ≥ 0.10).

##### Interaction between time and social network

The one-way ANOVA with T3-Pre difference score for subjective cognitive functioning as dependent variable and social network bins (i.e. based on the LSNS-6 score) as between-subject factor was significant, *F*(3,367) = 9.83, *p* < 0.001, $\eta_{p}^{2}$ = 0.074. Post-hoc independent samples* t*-tests with Bonferroni correction (*α* = 0.008) showed that participants with the lowest social support (bin 1, *M* = -0.52) differed significantly in subjective cognitive functioning difference score from participants with the second highest (bin 3, *M* = 0.32, *t*(178.72) = -4.65, *p* < 0.001, *d* = -0.66) and highest social support (bin 4, *M* = 0.14, *t*(158.91) = -4.01, *p* < 0.001,* d* = -0.56). Moreover, the difference score of participants with the second lowest social support (bin 2, *M* = -0.10) was significantly different from the score of participants with the second highest social support (bin 3, M = 0.32, *t*(181) = -2.67, *p* = 0.008, *d* = -0.40). Whereas participants in the two lowest social support bins showed decreases on T3 in subjective cognitive functioning compared to before the pandemic (measured retrospectively), subjective cognitive functioning increased for the two highest social support bins. This is visually presented in Supplementary Figure 1c. All other comparisons were not significant (*p* ≥ 0.019).

##### Interaction between time and presence of anxiety symptoms

The one-way ANOVA with T3-Pre difference score for subjective cognitive functioning as dependent variable and anxiety symptoms bins (i.e. based on the HADS score) as between-subject factor was significant, *F*(3,367) = 15.40, *p* < 0.001, $\eta_{p}^{2}$ = 0.11. Post-hoc independent samples *t*-tests with Bonferroni correction (*α* = 0.008) showed that participants with the highest anxiety symptoms (bin 4, *M* = -0.72) showed a significant negative difference score indicating a decrease in subjective cognitive functioning compared to participants with the lowest (bin 1, *M* = 0.31, *t*(157.55) = 6.34, *p* < 0.001, *d* = 0.93) and second lowest (bin 2, *M* = 0.095, *t*(161.96) = 5.03, *p* < 0.001, *d* = 0.72) anxiety symptoms, who even show a slightly positive difference score indicating an increase in subjective cognitive functioning. Moreover, participants with the highest anxiety symptoms (bin 4, *M* = -0.72) showed a significantly more negative difference score and thus a steeper decrease in subjective cognitive functioning compared to participants with the second highest anxiety symptoms (bin 3, *M* = -0.058, *t*(142) = 2.87, *p* = 0.005, *d* = 0.72). This is visually presented in Supplementary Figure 1d. All other comparisons were not significant (*p* ≥ 0.084).

To shortly summarize, declines in subjective cognitive functioning from before the pandemic (measured retrospectively) to T3, during the second peak of the pandemic, were accompanied by a high frequency of cognitive failures, depressive and anxiety symptoms and low social support.

#### Protective and risk factors and changes in subjective wellbeing

The same repeated measures analyses conducted on general subjective wellbeing as outcome showed significant main effects of frequency of cognitive failures (*F*(1, 349) = 13.03, *p* < 0.001, $\eta_{p}^{2}$ = 0.036), depressive symptoms (*F*(1, 349) = 37.00, *p* < 0.001, $\eta_{p}^{2}$ = 0.096), social network (*F*(1, 349) = 17.73, *p* < 0.001, $\eta_{p}^{2}$ = 0.048), resilience (*F*(1, 349) = 21.44, *p* < 0.001, $\eta_{p}^{2}$ = 0.058) and anxiety symptoms (*F*(1, 349) = 28.87, *p* < 0.001, $\eta_{p}^{2}$ = 0.076). As can be seen on Supplementary Figure 2, higher frequencies of cognitive failures, more depressive and anxiety symptoms and lower resilience, were related to lower overall subjective wellbeing scores. Again, this effect seemed to be the most pronounced for participants with the lowest scores on resilience (i.e. bin 1) and the highest scores on frequency of cognitive failures, depressive and anxiety symptoms (i.e., bin 4). Moreover, significant interaction effects between time and frequency of cognitive failures (*F*(3, 347) = 3.90, *p* = 0.009, $\eta_{p}^{2}$ = 0.033), time and depressive symptoms (*F*(3, 347) = 22.18, *p* < 0.001, $\eta_{p}^{2}$ = 0.16), time and resilience (*F*(3, 347) = 4.12, *p* = 0.007, $\eta_{p}^{2}$ = 0.034) and time and anxiety symptoms (*F*(3, 347) = 30.29, *p* < 0.001, $\eta_{p}^{2}$ = 0.21) were present. None of the other main effects and interactions were significant (*p* ≥ 0.075). Again, these interactions were further interpreted by comparing the T3-Pre difference score for cognitive functioning (and T1-Pre, T2-T1, T3-T2, T2-Pre and T3-T1 if the T3-Pre comparison does not allow us to interpret the interaction) between the different bins or levels of the covariate. In Supplementary Table 5 the mean T3-Pre difference scores for subjective wellbeing for each bin depending on the protective or vulnerability factor can be found.

##### Interaction between time and frequency of cognitive failures

The one-way ANOVA with the T3-Pre difference score for wellbeing as dependent variable and cognitive failures bins (i.e. based on the CFQ score) as between-subject factor was not significant, *F*(3,360) = 0.29, *p* = 0.83, $\eta_{p}^{2}$ = 0.002. Therefore, to further explore the interaction, one-way ANOVA’s with the T1-Pre (*F*(3,360) = 1.42, *p* = 0.24, $\eta_{p}^{2}$ = 0.012), the T2-T1 (*F*(3,360) = 0.24, *p* = 0.87, $\eta_{p}^{2}$ = 0.002), the T3-T2 (*F*(3,360) = 0.54, *p* = 0.65, $\eta_{p}^{2}$ = 0.005), the T2-Pre (*F*(3,360) = 0.55, *p* = 0.65, $\eta_{p}^{2}$ = 0.005) and the T3-T1 (*F*(3,360) = 1.03, *p* = 0.38, $\eta_{p}^{2}$ = 0.008) difference scores as dependent variable and cognitive failures bins as between-subject factor were conducted as well. However, none of these comparisons reached significance implying that they could not aid in further explaining the significant interaction in the repeated measures ANOVA. Even though this interaction between time and cognitive failures proved to be significant in the repeated measures ANOVA, none of the post hoc tests could explain this significant interaction and visually (see Supplementary Figure 2a) the interaction is not clearly observable in the data as well.

##### Interaction between time and presence of depressive symptoms

The one-way ANOVA with the T3-pre difference score for wellbeing as dependent variable and depressive symptoms bins (i.e. based on the GDS-15 score) as between-subject factor was significant, *F*(3,367) = 22.17, *p* < 0.001, $\eta_{p}^{2}$ = 0.15. Post-hoc independent samples *t*-tests with Bonferroni correction (*α* = 0.008) showed that participants with the highest depressive symptoms (bin 4, *M* = -12.16) had a significantly more negative difference score, and thus a steeper decrease in wellbeing since before the pandemic, measured retrospectively, compared to participants in the lower bins (bin 1, *M* = -2.81, *t*(118.88) = 7.03, *p* < 0.001, *d* = 1.08; bin 2, *M* = -3.46, *t*(159.12) = 5.56, *p* < 0.001, *d* = 0.86; bin 3, *M* = -4.97, *t*(126) = 3.42, *p* < 0.001, *d* = 0.64). This is visually presented in Supplementary Figure 2b. All other comparisons were not significant (*p* ≥ 0.23).

##### Interaction between time and resilience

The one-way ANOVA with T3-pre difference score for wellbeing as dependent variable and resilience bins (i.e. based on the BRS score) as between-subject factor was significant, *F*(3,367) = 3.02, *p* = 0.030, $\eta_{p}^{2}$ = 0.024, but the post-hoc independent samples *t*-tests failed to reach significance after Bonferroni correction (*α* = 0.008). Therefore, to further explore the significant interaction between time and resilience, one-way ANOVAs with the T1-pre (*F*(3,367) = 4.46, *p* = 0.004, $\eta_{p}^{2}$ = 0.035), T2-T1 (*F*(3,367) = 0.50, *p* = 0.68, $\eta_{p}^{2}$ = 0.004) and T3-T2 (*F*(3,367) = 1.67, *p* = 0.17, $\eta_{p}^{2}$ = 0.013) difference scores for wellbeing as dependent variable and resilience bins as between-subject factor were conducted as well. Post-hoc independent samples *t*-tests with Bonferroni correction (*α* = 0.008) for the significant ANOVA with difference score T1-pre showed that participants with the lowest resilience (bin 1, *M* = -8.68) had a significantly steeper decrease in subjective wellbeing from before the pandemic, measured retrospectively, to T1 compared to participants with the second lowest (bin 2, *M* = -4.00, *t*(135.64) = -3.70,* p* < 0.001, *d* = -0.48) and highest resilience (bin 4, *M* = -5.16, *t*(207.87) = -2.81,* p* = 0.005, *d* = -0.37). This is visually presented in Supplementary Figure 2c. All other comparisons were not significant (*p* ≥ 0.012).

##### Interaction between time and the presence of anxiety symptoms

The one-way ANOVA with T3-pre difference score for wellbeing as dependent variable and anxiety symptoms bins (i.e. based on the HADS score) as between-subject factor was significant, *F*(3,367) = 42.96, *p* < 0.001, $\eta_{p}^{2}$ = 0.26. Post-hoc independent samples *t*-tests with Bonferroni correction (*α* = 0.008) showed that participants with the highest anxiety symptoms (bin 4, *M* = -13.61) had a significantly more negative difference score, and thus a steeper decrease in wellbeing since before COVID measured retrospectively, compared to participants from the lower bins (bin 1, *M* = -1.43, *t*(145.17) = 9.53, *p* < 0.001, *d* = 1.41; bin 2, *M* = -2.30, *t*(152.23) = 8.75, *p* < 0.001, *d* = 1.27; bin 3, *M* = -5.82, *t*(142) = 4.39, *p* < 0.001, *d* = 0.76). Moreover, participants with the second highest anxiety symptoms (bin 3, *M* = -5.82) had a significantly steeper decrease in wellbeing since the pandemic compared to participants with the lowest (bin 1, *M* = -1.43, *t*(75.56) = 3.04, *p* = 0.003, *d* = 0.59) and second lowest anxiety symptoms (bin 2, *M* = -2.30, *t*(176) = 2.67, *p* = 0.008, *d* = 0.44). This is visually presented in Supplementary Figure 2d. All other comparisons were not significant (*p* ≥ 0.35).

To shortly summarize, declines in subjective wellbeing from before the pandemic (measured retrospectively) to T3, during the second peak of the pandemic, were accompanied by high depressive and anxiety symptoms and by low resilience, although the latter was clearly less prominent.

## Discussion

Earlier studies showed a significant impact of the initial stages of the pandemic on wellbeing and cognitive functioning of older adults (e.g.,^1^). Studies on the longer-term impact of the pandemic on older adults are still scarce and showed mixed findings. Based on earlier studies we expected a sustained impact of the pandemic on wellbeing and subjective cognitive functioning, with wellbeing and subjective cognitive functioning potentially fluctuating with the severity of the pandemic. To unravel this, we assessed the longer-term impact of the pandemic on subjective cognitive functioning and wellbeing by following up the sample of older adults from the first wave of our COVID-19 survey study which was collected just after the first peak of the pandemic (i.e., T1;^1^). This group of older adults was then re-assessed in different phases of the pandemic which were less and more severe (i.e., T2 and T3, respectively).

Based on our findings it seems that the pandemic had a long-lasting impact on older adults, without fully recovering from this extreme stressor. Regarding *subjective cognitive functioning*, results were mixed. Self-reported general subjective cognitive functioning was slightly better on T3 than on T2, reaching similar ratings on T3 as before the pandemic, suggesting recovery of general subjective cognitive functioning. Nevertheless, effect sizes were overall small. However, when assessing subjective cognitive functioning in more detail, the percentage of participants reporting more problems with subdomains of cognitive functioning (e.g., problems with recalling) since the past month, dramatically increased towards our last measurement moment and for all cognitive subdomains. As COVID-19 cases increased again and governmental regulations became more strict in Belgium on T3, with the start of a new lockdown in November 2020, stress and worry could have increased, resulting in more subjective cognitive complaints. Indeed, when looking at the scores on the CFQ (see Table 6 for mean CFQ scores, the Supplementary results and Supplementary Figure 3), we also noticed a linear increase in cognitive failures over the measurement moments. This could be partially explained by having more opportunities for cognitive failures in daily life situations, such as the supermarket or social interactions, in less severe pandemic times compared to the most strict lockdown (i.e., T1). The supermarket item was one of the items of the CFQ with the highest number of participants indicating “not applicable” throughout the measurement moments, indicating that a part of the older adults did not visit the supermarket during our study. However, cognitive failures on T3 (the second lockdown) were still more frequent than on T2 (a period without any restrictions). Similarly, da Silva Castanheira et al.^7^ found that pandemic related worry significantly predicted declines in objective cognitive functioning. Interestingly, this pattern was not visible in the general cognitive functioning ratings, suggesting that older adults base their ratings of general subjective cognitive functioning, measured with only one item, on more than problems in cognitive subdomains alone, or tend to overestimate their overall cognitive functioning level^49^. Alternatively, we also cannot exclude the possibility that older adults overestimated the frequency of cognitive failures. Of note, decreases in subjective cognitive functioning in the current study were especially prominent for problems with recalling. Other studies have also already identified recalling as impacted by the pandemic, linking it to sleep-related problems^50^.

For *wellbeing*, our results were in line with other research, showing longer-term fluctuations in wellbeing over the three measurement moments mimicking fluctuations in pandemic severity^7,17^. However, our study showed that levels of wellbeing during the last phase (T3) were still significantly lower than before the pandemic, measured retrospectively, indicating a sustained impact of the pandemic. Overall, effect sizes were large, showing the sustained impact of the pandemic on the mental health of older adults. One possible explanation for this negative longer-term impact of the pandemic on older adults can be found within the strength and vulnerability integration (SAVI) model. This theoretical model poses that older adults normally make great use of coping strategies to maintain a high emotional wellbeing. However, when faced with extreme (and longer-term) stressor situations like the pandemic, it can become difficult to maintain and regulate high levels of wellbeing^51^.

Importantly, we observed that the impact of the pandemic varied between older adults. When looking at possible protective and vulnerability factors, especially higher depressive symptoms (measured on T1) and anxiety symptoms (measured on T3) seemed to be important risk factors for declines in subjective cognitive functioning and subjective wellbeing, as indicated by the large effect sizes. Remarkably, for *cognitive functioning*, only participants with the lowest depressive symptoms (measured on T1), the lowest and second lowest anxiety symptoms (measured on T2) and the lowest and second lowest social support (measured on T1) showed declines in subjective cognitive functioning. This is in line with other studies, linking depressive and anxiety symptoms^30^ and low social support^26,27^ to declines in cognitive functioning. All other participants in this study showed no differences or even slight increases in cognitive functioning ratings compared to before the pandemic. Regarding *wellbeing*, all participants showed declines in general subjective wellbeing at T3 compared to before the pandemic. However, declines were the strongest for those participants with the highest depressive and anxiety symptoms, making them important vulnerability factors in this extreme stressor situation. The association between protective and vulnerability factors such as cognitive failures and resilience and changes in subjective cognitive functioning and wellbeing was rather small.

The Socioemotional Selectivity theory^33^ proposes that older adults can maintain high levels of wellbeing due to their social relationships, buffering them against negative impacts of life experiences. In addition, the Coping, Appraisal, and Resilience in Aging model predicts that older adults are able to successfully cope with stressor situations by viewing them as less stressful or problematic^52^. However, our results show that extreme stressor situations such as the pandemic, that restrict social contacts, might diminish this protective buffer, impacting older adults’ wellbeing. Based on our results we propose that the focus of the biopsychosocial model^31,32^ on the interaction between multiple psychological and social factors makes this model well-suited to explain the impact of the pandemic on mental health, as other researchers have also already shown in the general population^53^.

We need to address some limitations of this study. First of all, as is often the case for research conducted during the pandemic, this study lacked an unbiased pre-pandemic assessment, as discussed in the paper of the first measurement moment of this study^1^. Previous research already showed that especially cognitive functioning is more prone to overestimation, particularly in older adults^54^. In addition, an online COVID survey in young adults showed a risk for underestimating changes in mental and physical health from before the pandemic, when assessing them using retrospective questions^55^. However, this longitudinal design enabled us to study the impact of the pandemic on older adults in more detail, by re-assessing these questions in different phases of the pandemic. By comparing ratings of wellbeing and cognitive functioning between periods with different severity levels of the pandemic, the impact of the pandemic itself on these outcome measures could be studied in more detail. However, we cannot exclude the possibility that observed changes in wellbeing and subjective cognitive functioning alternatively were the result of other confounding variables that covaried with the changes in the COVID-19 context or age-related changes, prohibiting us to make causal claims. Second, the online format of the study and the reliance on self-reports could have led to a bias, not reaching all older adults and possibly over- or underestimating the impact of the pandemic. For a more detailed discussion, we refer to De Pue et al.^1^. This is especially important for ratings of cognitive functioning. As this was a self-report study, only subjective cognitive functioning was measured. However, research shows mixed findings about the relation between subjective and objective cognitive functioning, especially in those adults with depressive symptoms^56^. Studies on the impact of the pandemic on objective cognitive functioning of older adults would be needed to shed more light on pandemic related changes in cognitive function in this age group. Third, the sample of older adults that took part in all measurement moments was even more homogenous than the sample of participants from T1: almost all older adults of this longitudinal study were still in very good health, and especially relatively younger older adults participated in the study. Moreover, participants who dropped out showed some different demographic characteristics (e.g. they were in general older, cf. Supplementary Table 1). In addition, these participants reported a significantly lower subjective wellbeing, more depressive symptoms, a lower social support and a lower resilience (cf. Supplementary Tables 3 and 4). This makes us cautious to generalize the findings of this study to the whole older adult population, as a part of this population who already reported lower wellbeing dropped out of the study. More studies in more heterogeneous samples of older adults could help in further unraveling the long-term impact of the pandemic on older adults. In addition, in this study we used a broad age range of older adults. However, recent studies show differences in the impact of the pandemic on young-old versus old-old adults^57,58^. Finally, some methodological limitations of this study need to be discussed. Not all protective and vulnerability factors were measured at the same measurement moment, whereas some were measured on each measurement moment. Ideally, we would have measured anxiety symptoms on T1 as well, instead of T3, since levels of this risk factor could have been different depending on the phase of the pandemic. Depressive symptoms were measured on each measurement moment, but did not differ significantly over time (cf. Supplementary results). However, frequency of cognitive failures (as assessed with the CFQ) did increase across the measurement moments, as explained above. Our full data set, including variables that were outside the scope of the current study such as coping, activity level and sleep, is openly accessibly on OSF (https://osf.io/vfwus/), allowing other researchers to further scrutinize the dataset. Especially sleep seems to be an important predictor of changes in mental health over the course of the pandemic^59^.

To summarize, based on our study it seems that the pandemic had a long-term impact on the cognitive functioning and wellbeing of older adults. Especially depressive and anxiety symptoms put a subgroup of older adults at high risk for prominent declines in our outcome measures. Studying and investing in these risk factors will be crucial to decrease the sustained impact of COVID-19 on these older adults and to perhaps prevent declines in wellbeing and cognitive functioning when faced with other extreme stressor situations in the future. Even though older adults are frequently seen as resilient, this study shows the increasing importance of monitoring mental health of older adults as extreme stressors develop across time. Regular psychological counseling via telephone or online could support older adults during this stressing periods^60,61^. Over the course of the pandemic, more and more researchers focused on the effects of the pandemic on mental health of older adults. However, an increasing number of research, including the current study, shows the emergence of cognitive health issues as well. Anxiety (as shown here) and worry (e.g.^6^) negatively impact subjective cognitive functioning in older adults. Finding ways to diminish such effects on cognitive functioning, for example by teaching people how to cope with extreme stressors^62^, can be especially important during extremely stressing times. In addition, neuropsychological counseling, via telephone or online interventions, could be worthy of exploration^63,64^. Moreover, as social network seems to be crucial to prevent further cognitive declines^2^-^4^ and mental health problems, maintaining social engagement of older adults, even in times of social distancing, should become an important aim.

## Supplementary Information

Supplementary Information.

## Data Availability

The anonymized, raw data that support the findings of this study are available in the Open Science Framework (OSF, https://osf.io/vfwus/).

## Code availability

The SPSS code used to generate results that are reported in the paper is available upon request, by contacting the corresponding author (sarah.depue@kuleuven.be).
