# Supplementary material for: The longer-term impact of the COVID-19 pandemic on wellbeing and subjective cognitive functioning of older adults in Belgium
Source: Sci Rep. 2023 Jun 15;13:9708. doi: 10.1038/s41598-023-36718-9 (PMC10272225; doi:10.1038/s41598-023-36718-9)
Supplement: Supplementary file 1 — Supplementary Information. [file 41598_2023_36718_MOESM1_ESM.pdf]

The longer-term impact of the COVID-19 pandemic on wellbeing and subjective cognitive functioning of older adults in Belgium

Supplementary Information

Sarah De Pue*, Céline Gillebert, Eva Dierckx & Eva Van den Bussche

*Corresponding author

E-mail: sarah.depue@kuleuven.be

Supplementary Table 1

Overview of participants' demographic and general characteristics measured on the different measurement moments.

			Final sample (*n*=371, 72y)		Drop-outs (*n*=269, 75y)	
			N	%	N	%
Age Category (T1)	65-69		154	41.5%	94	34.9%
	70-74		129	34.8%	58	21.6%
	75-79		54	14.6%	33	12.3%
	80-84		22	5.9%	36	13.4%
	85-89		10	2.7%	28	10.4%
	≥90		2	0.5%	20	7.4%
Gender (T1)	Male		172	46.4%	91	33.8%
	Female		199	53.6%	178	66.2%
Nationality (T1)	Belgian		368	99.2%	264	98.1%
	Dutch		3	0.8%	2	0.7%
	Moroccan		0	-	2	0.7%
	German		0	-	1	0.4%
Living situation (T1)	Own or partner's home		344	92.7%	174	64.7%
	Care facility	Nursing home	3	0.8%	50	18.6%
		Assisted living facility	24	6.5%	43	16%
	Other		0	-	2	0.7%
Number of cohabitants (T1)	0		115	31.0%	64	23.8%
	1		217	58.5%	128	47.6%
	2 or more		39	10.5%	77	28.6%
Highest educational level (T1)	No secondary school diploma		20	5.4%	61	22.7%
	Secondary school diploma		93	25.1%	88	32.7%
	University or high school degree		233	62.8%	98	36.4%
	Other		25	6.7%	22	8.2%
Work situation (T1)	Retired		357	96.2%	253	94.1%
	Employed		6	1.6%	8	3.0%
	Other (e.g., disabled or retired with part-time job)		8	2.2%	8	2.9%
Monthly individual net income (in €) (T1)	0-500		13	3.5%	18	6.7%
	501-1000		7	1.9%	15	5.6%
	1001-1500		83	22.4%	78	29%
	1501-2000		129	34.8%	60	22.3%
	2001-2500		83	22.4%	26	9.7%
	2501-3000		21	5.7%	15	5.6%
	3001-3500		8	2.2%	1	0.4%
	3501-4000		3	0.8%	3	1.1%
	Above 4000		7	1.9%	1	0.4%
	I can't or do not want to answer this question		17	4.6%	52	19.3%
Medical history (T1)	Parkinson's disease		1	0.3%	6	2.2%

	Dementia		0	-	7	2.6%
	Stroke		3	0.8%	6	2.2%
	Diabetes		30	8.1%	23	8.6%
	Epilepsy		1	0.3%	2	0.7%
	None of the above		336	90.6%	227	84.4%
COVID-19 contagion of participant	T1	No	357	96.2%	256	95.2%
		Yes, confirmed with test	2	0.5%	5	1.9%
		Yes, but not confirmed with test	12	3.2%	8	3.0%
	T2	No	357	96.2%	-	-
		Yes, confirmed with test	2	0.5%	-	-
		Yes, but not confirmed with test	12	3.2%	-	-
	T3	No	359	96.8%	-	-
		Yes, confirmed with test	3	0.8%	-	-
		Yes, but not confirmed with test	9	2.4%	-	-
COVID-19 contagion of at least one close relative or friend	T1	No	314	84.6%	228	84.8%
		Yes, confirmed with test	25	6.7%	18	6.7%
		Yes, but not confirmed with test	32	8.6%	23	8.6%
	T2	No	294	79.2%	-	-
		Yes, confirmed with test	39	10.5%	-	-
		Yes, but not confirmed with test	38	10.3%	-	-
	T3	No	275	74.1%	-	-
		Yes, confirmed with test	80	21.6%	-	-
		Yes, but not confirmed with test	16	4.3%	-	-
Vaccination readiness (T3)	No		20	5.4%	-	-
	Yes		351	94.6%	-	-
Week when survey was completed	T1	19/5/2020-25/5/2020	198	53.4%	87	32.3%
		26/5/2020-1/6/2020	63	17.0%	43	16.0%
		2/6/2020-8/6/2020	77	20.8%	63	23.4%
		9/6/2020-15/6/2020	19	5.1%	51	19.0%
		16/6/2020-22/6/2020	14	3.7%	25	9.3%
	T2	16/6/2020-22/6/2020	195	52.6%	-	-
		23/6/2020-29/6/2020	65	17.5%	-	-
		30/6/2020-6/7/2020	72	19.4%	-	-
		7/7/2020-13/7/2020	26	7.0%	-	-
		14/7/2020-16/7/2020	13	3.5%	-	-
	T3	9/12/2020-15/12/2020	273	73.6%	-	-
		16/12/2020-22/12/2020	85	22.9%	-	-
		23/12/2020-29/12/2020	8	2.2%	-	-
		30/12/2020-18/1/2021	5	1.3%	-	-

Supplementary Table 2

Overview of participants' number of contacts during the past week measured on T1, T2 and T3.

			T1	T2	T3
Number of contacts during the past week (excluding cohabitants)	In real life outside	0	34	15	54
		1	28	13	39
		2	52	35	74
		3 or 4	117	92	117
		5 to 8	94	118	64
		+9	45	98	23
	In real life inside	0	168	60	75
		1	55	41	113
		2	60	47	97
		3 or 4	63	111	64
		5 to 8	15	80	14
		+9	9	32	8
	By telephone	0	16	12	14
		1	13	19	23
		2	42	41	53
		3 or 4	129	126	128
		5 to 8	102	112	100
		+9	69	61	53
	By internet	0	35	39	35
		1	15	11	18
		2	34	36	37
		3 or 4	87	93	86
		5 to 8	86	94	92
		+9	113	98	103

Supplementary Table 3

Means (standard deviations) of reported subjective cognitive functioning and wellbeing on PRE and T1, percentages of participants reporting more problems with specific subdomains of subjective cognitive functioning since the past month and effect sizes for participants that are included in the study vs. those who dropped out before the third wave.

		Pre			T1		
		Mean (*SD*) final sample	Mean (*SD*) drop-outs	Final sample vs. drop-outs	Mean (*SD*) final sample	Mean (*SD*) drop-outs	Final sample vs. drop-outs
Subjective cognitive functioning	Decrease in general subjective cognitive functioning	-	-	-	7%	9%	n.s.
	Problems with remembering	-	-	-	7%	10%	n.s.
	Problems with concentrating	-	-	-	12%	13%	n.s.
	Problems with doing two things at the same time	-	-	-	5%	7%	n.s.
	Problems with recalling	-	-	-	9%	11%	n.s.
	Problems with forgetfulness	-	-	-	8%	12%	n.s.
Wellbeing (PWI-A)	General subjective wellbeing	79.50 (9.50)	76.01 (12.99)	***, *d*=.32	72.96 (12.78)	67.84 (16.72)	***, *d*=.33
	General life satisfaction	79.84 (11.27)	76.06 (15.61)	***, *d*=.29	71.02 (17.07)	65.32 (20.71)	***, *d*=.31
	Standard of living	81.37 (11.22)	77.51 (15.58)	***, *d*=.29	78.89 (13.46)	71.45 (20.58)	***, *d*=.44
	Health	77.79 (12.28)	72.34 (18.63)	***, *d*=.36	75.15 (14.75)	69.15 (19.84)	***, *d*=.35
	Achieving in life	79.92 (12.25)	76.32 (15.22)	**, *d*=.27	76.42 (15.19)	71.93 (18.47)	**, *d*=.27
	Relationships	79.76 (14.03)	76.94 (16.29)	*, *d*=.19	72.94 (19.19)	69.48 (21.32)	n.s.
	Safety	81.78 (10.00)	79.48 (13.87)	*, *d*=.20	71.94 (16.34)	68.33 (20.87)	*, *d*=.20
	Community connectedness	78.01 (13.19)	75.65 (15.38)	n.s.	68.01 (18.00)	63.53 (21.95)	**, *d*=.23
	Future security	77.87 (11.67)	74.09 (15.75)	***, *d*=.28	67.39 (16.73)	63.83 (20.38)	*, *d*=.19

Note. PWI-A = Personal Wellbeing Index – Adults total score; *** $p<.001$, ** $p<.010$, * $p<.050$, n.s. = non-significant.

Supplementary Table 4

Mean scores and standard deviations for the protective and vulnerability factors included in the current study on T1 and effect sizes for participants that are included in the study vs. those who dropped out before the third wave.

	Range	Final sample		Drop-outs		Final sample vs. drop-outs
		n	Mean (*SD*)	*n*	Mean (*SD*)	
Cognitive failures (CFQ)	0 - 100	364	22.61 (11.25)	259	22.18 (13.05)	n.s.
Depressive symptoms (GDS-15)	0 - 15	371	2.60 (2.66)	269	3.56 (3.37)	***, *d*=-.32
Social network (LSNS-6)	0 - 30	371	17.54 (5.22)	265	15.08 (5.92)	***, *d*=.45
Resilience (BRS)	1 - 5	371	3.40 (0.65)	262	3.25 (0.69)	**, *d*=.23

Note. CFQ = Cognitive Failures Questionnaire total score; GDS-15 = Geriatric Depression Scale-15 total score; LSNS-6 = Lubben Social Network Scale-6 total score; BRS = Brief Resilience Scale mean score; HADS = Hospital Anxiety and Depressive symptoms sum score on the anxiety items; *** $p<.001$, ** $p<.010$, n.s. = non-significant.

Supplementary Table 5

Mean difference scores (T3-Pre) and standard deviations for the different bins of the significant protective and vulnerability factors for changes in subjective cognitive functioning and subjective wellbeing over time.

	Protective or vulnerability factor	Bin 1		Bin 2		Bin 3		Bin 4	
		n	Mean (*SD*)	*n*	Mean (*SD*)	*n*	Mean (*SD*)	*n*	Mean *(SD)*
Subjective cognitive functioning	Cognitive failures	92	-0.13 (1.10)	99	0.051 (1.06)	88	0.00 (0.98)	85	0.26 (1.51)
	Depressive symptoms	163	0.055 (0.94)	80	0.28 (1.07)	42	0.24 (0.69)	86	-0.78 (1.50)
	Social network	103	-0.52 (1.46)	102	-0.10 (1.09)	81	0.32 (1.00)	85	0.14 (0.76)
	Anxiety symptoms	101	0.31 (0.88)	126	0.095 (0.98)	52	-0.058 (1.36)	92	-0.72 (1.30)
Subjective wellbeing	Cognitive failures	92	-5.59 (9.35)	99	-5.38 (9.29)	88	-4.53 (8.60)	85	-5.83 (11.35)
	Depressive symptoms	163	-2.81 (6.85)	80	-3.46 (8.78)	42	-4.97 (10.90)	86	-12.16 (11.29)
	Resilience	114	-7.56 (12.25)	35	-3.35 (7.89)	101	-4.53 (8.60)	121	-4.58 (7.87)
	Anxiety symptoms	101	-1.43 (6.33)	74	-2.30 (7.39)	112	-5.82 (9.39)	63	-13.62 (10.67)

Supplementary Figure 1

The association between protective and vulnerability factors and changes in subjective cognitive functioning.

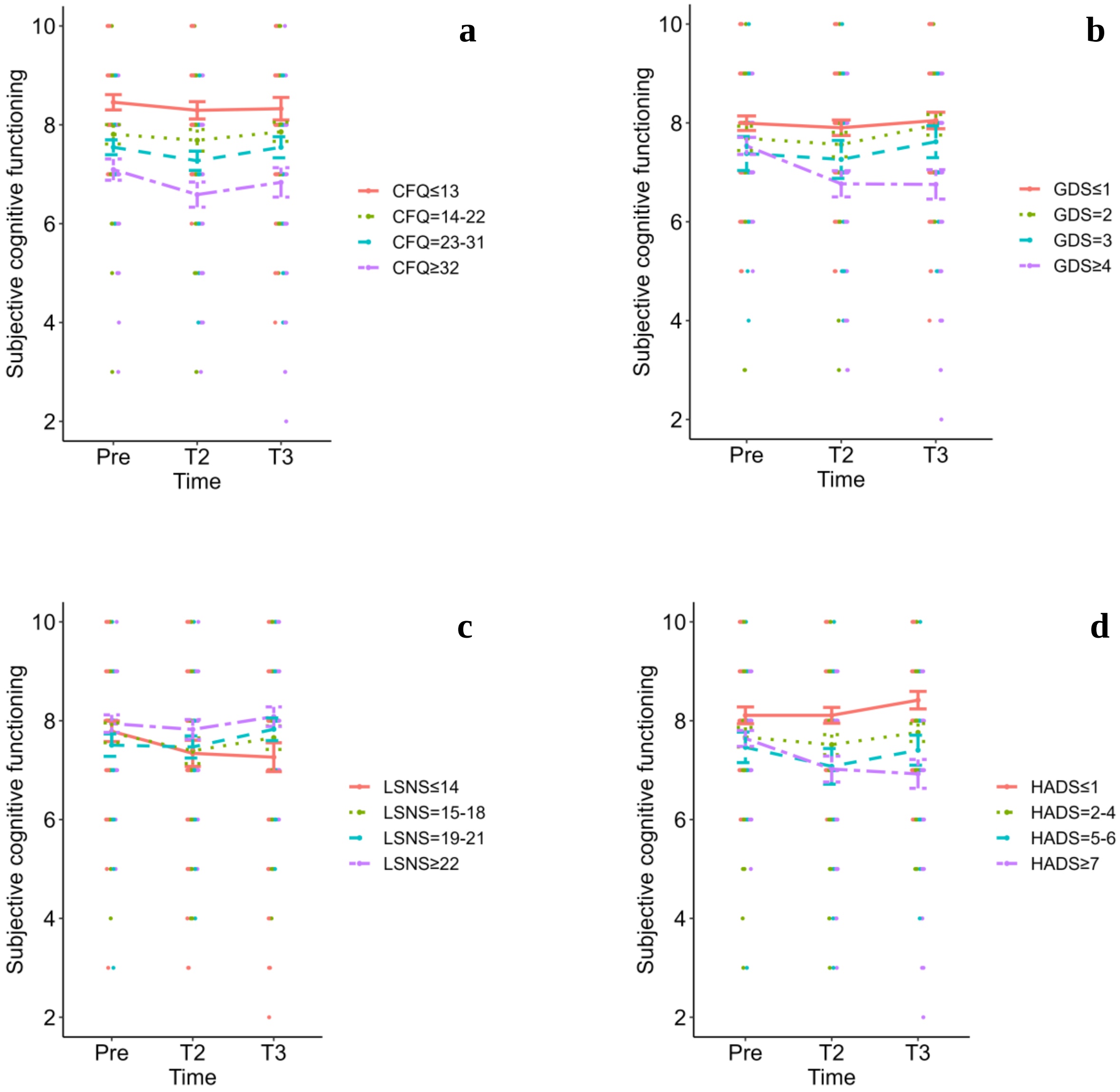

Note. Panel a shows the frequency of cognitive failures, measured with CFQ (Cognitive Failures Questionnaire), and changes in subjective cognitive functioning; Panel b shows depressive symptoms, measured with GDS-15 (Geriatric Depression Scale-15), and changes in subjective cognitive functioning; Panel c shows social network, measured with LSNS-6 (Lubben Social Network Scale-6), and changes in subjective cognitive functioning and Panel d shows anxiety symptoms, measured with HADS (the anxiety items of the Hospital Anxiety and Depressive Symptoms questionnaire), and changes in subjective cognitive functioning.

Supplementary Figure 2

The association between protective and vulnerability factors and changes in subjective wellbeing.

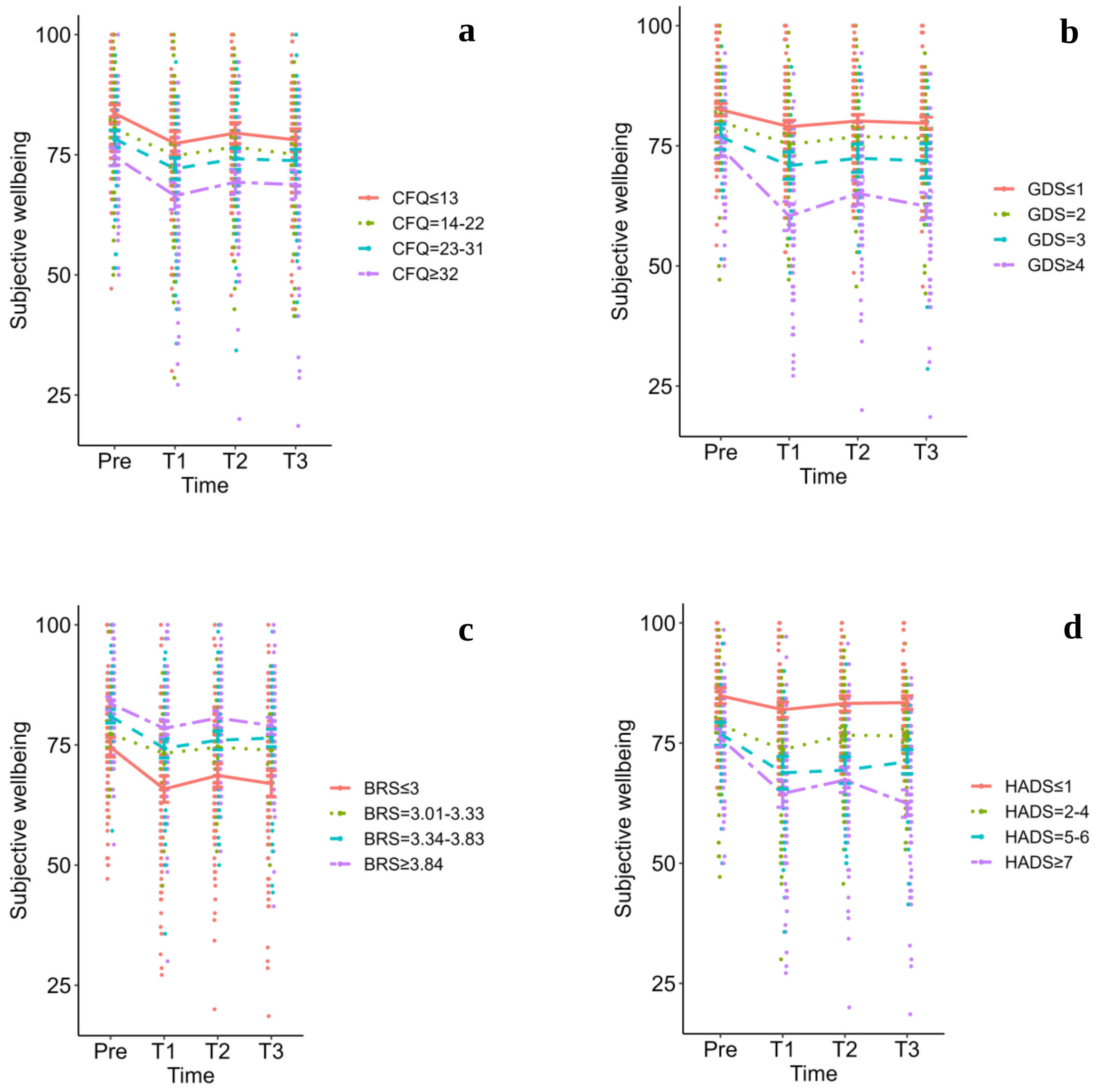

Note. Panel a shows the frequency of cognitive failures, measured with CFQ (Cognitive Failures Questionnaire), and changes in subjective wellbeing; Panel b shows depressive symptoms, measured with GDS-15 (Geriatric Depression Scale-15), and changes in subjective wellbeing; Panel c shows resilience, measured with BRS (Brief Resilience Scale), and changes in subjective wellbeing and Panel d shows anxiety symptoms, measured with HADS (the anxiety items of the Hospital Anxiety and Depressive Symptoms questionnaire), and changes in subjective wellbeing.

Supplementary results

In these exploratory analyses, the effect of the different phases of the pandemic on depressive symptoms and frequency of cognitive failures was studied. Repeated measures ANOVAs were conducted with time as within-subject factor and self-reported ratings of depressive symptoms on a scale from 0-15, and frequency of cognitive failures rated on a scale from 0-100 (both measured on T1, T2 and T3) as outcome variables. Post-hoc paired samples t-tests with Bonferroni correction were used to compare scores on the outcome measures between different measurement moments.

In addition, repeated measures ANOVAs were used with time as within-subject factor and adding between-subject factors and covariates, to study the association between protective and vulnerability factors and these observed changes in subjective cognitive functioning and wellbeing. Gender, living assisted or not and living alone or not were added as between-subject factors, and age and monthly net income were added as covariates. Furthermore, social network and resilience were added as covariates, as they were potential protective and vulnerability factors.

Changes in depressive symptoms and reported cognitive failures over the course of the pandemic

Table 3 shows the means scores for depressive symptoms and cognitive failures over time.

Changes in depressive symptoms over time

The repeated measures ANOVA with time as within-subject factor showed no significant main effect of time ($F(2,369)=1.60$, $p=.20$, $\eta_p^2=.009$). The frequency of depressive symptoms did not change over the course of the pandemic.

Changes in reported cognitive failures over time.

The repeated measures ANOVA with time as within-subject factor showed a significant main effect of time ($F(2,357)=47.19$, $p<.001$, $\eta_p^2=.21$). Post-hoc paired samples *t*-tests with Bonferroni correction ($\alpha=.017$) showed that cognitive failures were significantly more frequent on T2 ($M=24.50$) compared to T1 ($M=22.61$), $t(360)=-4.85$, $p<.001$, $d=-0.26$. Cognitive failures on T3 ($M=26.85$) were significantly more frequent than on T1 ($M=22.61$), $t(361)=-9.80$, $p<.001$, $d=-0.52$, and T2 ($M=24.50$),

$t(364)=-5.76$, $p<.001$, $d=-0.30$. The frequency of cognitive failures thus linearly increased over the course of the pandemic.

Supplementary Figure 3

Changes in depressive symptoms (a) and frequency of cognitive failures (b) over the course of the pandemic.

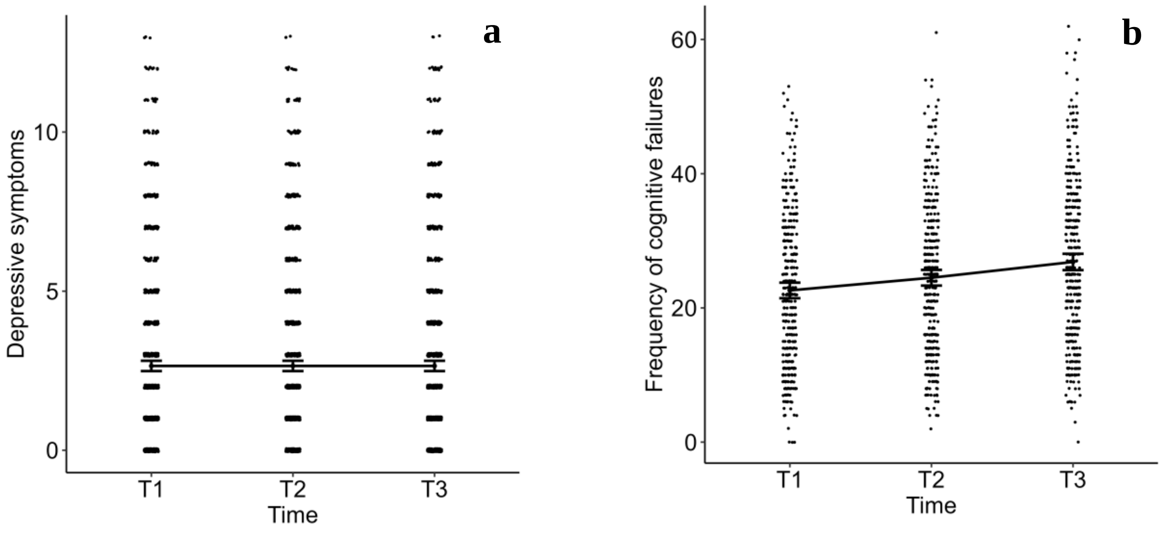

The association between protective and vulnerability factors and the changes in depressive symptoms and cognitive failures

Table 6 shows the means scores for the protective and vulnerability factors over time.

Moderators and changes in depressive symptoms.

The repeated-measures ANOVA with time as within-subject factor, gender, living assisted or not and living alone or not as between-subject factors and age, monthly net income, social network and resilience as covariates showed a significant main effect of social network ($F(1,359)=35.02$, $p<.001$, $\eta_p^2=.089$), resilience ($F(1,359)=107.17$, $p<.001$, $\eta_p^2=.23$) and living alone or not ($F(1,359)=9.56$, $p=.002$, $\eta_p^2=.026$). Moreover, a significant interaction between living alone or not and assisted living or not was observed ($F(1,359)=4.86$, $p=.028$, $\eta_p^2=.013$). As visible on Supplementary Figure 4, participants with a lower social support and lower resilience showed overall higher depressive

symptoms. Moreover, participants living alone (*M*=3.66) showed overall higher depressive symptoms compared to participants not living alone (*M*=2.20; *t*(160.07)=-4.70, *p*<.001, *d*=-0.61). This effect was larger for older adults living in assisted care (*t*(23.13)=-2.93, *p*=.008, *d*=-1.07) than for older adults not living in assisted care (*t*(138.72)=-3.82, *p*<.001, *d*=-0.53). All other main and interaction effects were not significant (all *p*>.087).

Supplementary Figure 4

The association between protective and vulnerability factors and changes in depressive symptoms.

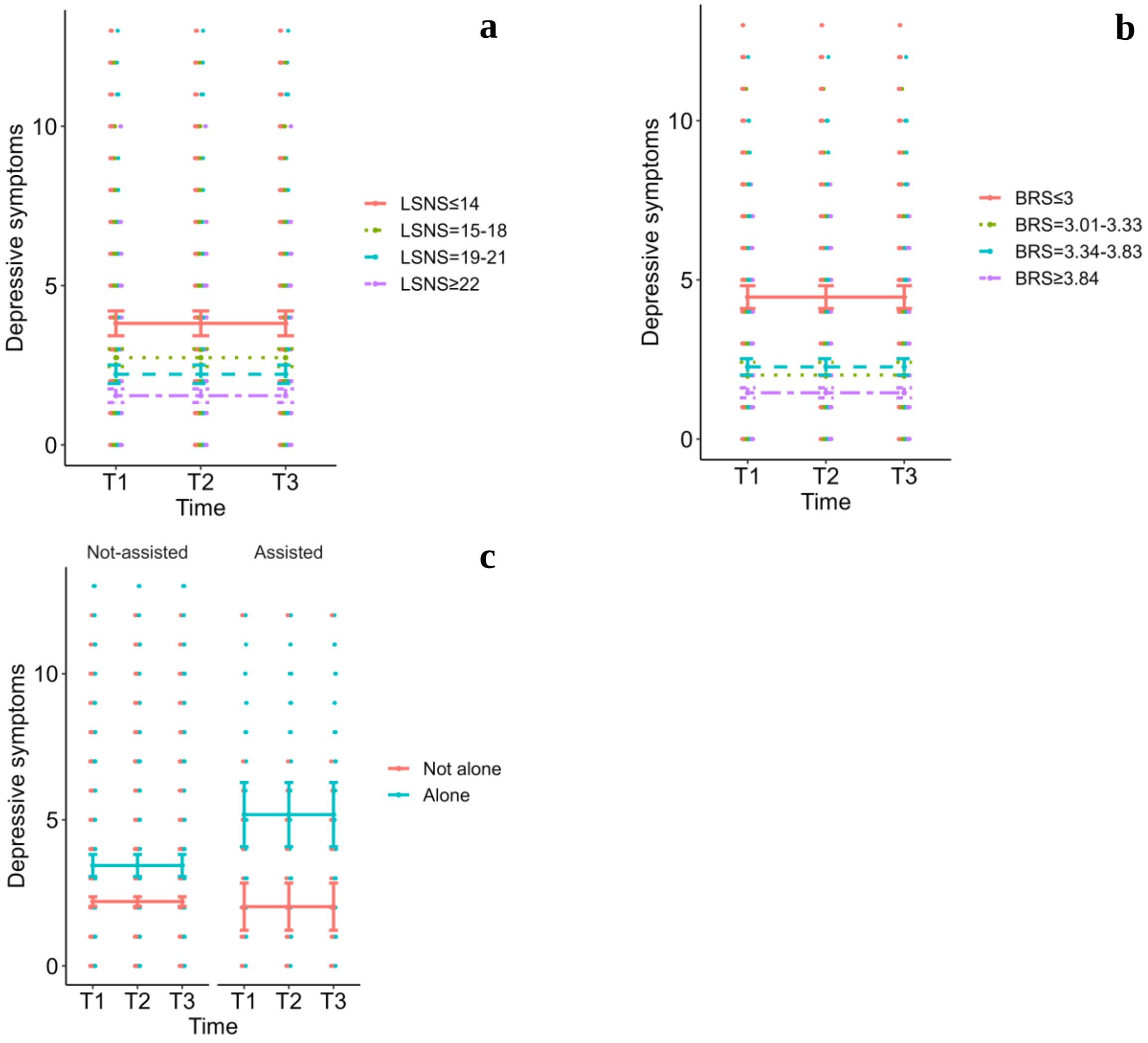

Note. Panel a shows social network, measured with LSNS-6 (Lubben Social Network Scale-6), and changes in depressive symptoms; Panel b shows resilience, measured with BRS (Brief Resilience Scale), and changes in depressive symptoms; Panel c shows the interaction between Living alone or not and Living assisted or not, and changes in depressive symptoms.

Moderators and changes in frequency of cognitive failures.

The repeated-measures ANOVA with time as within-subject factor, gender, living assisted or not and living alone or not as between-subject factors and age, monthly net income, social network and resilience as covariates showed a significant main effect of age (F(1,347)=4.34, p=.038, η_p^2=.012), monthly net income (F(1,347)=4.02, p=.046, η_p^2=.011), resilience (F(1,347)=36.25, p<.001, η_p^2=.095), and gender (F(1,347)=6.49, p=.011, η_p^2=.018). Moreover, a significant interaction between gender and assisted living or not was observed (F(1,347)=6.41, p=.012, η_p^2=.018). As visible on Supplementary Figure 5, younger participants, participants with a lower resilience and a lower income showed an overall higher frequency of cognitive failures. Moreover, female participants not in assisted care (M=26.08) showed an overall higher frequency of cognitive failures compared to male participants not in assisted care (M=23.68; t(329.90)=-2.15, p=.033, d=-0.23), whereas there was no significant difference between male (M=26.39) and female participants in assisted care (M=19.92; t(15.84)=1.44, p=.17). All other main and interaction effects were not significant (all p>.067).

Supplementary Figure 5

The association between protective and vulnerability factors and changes in the frequency of cognitive failures.

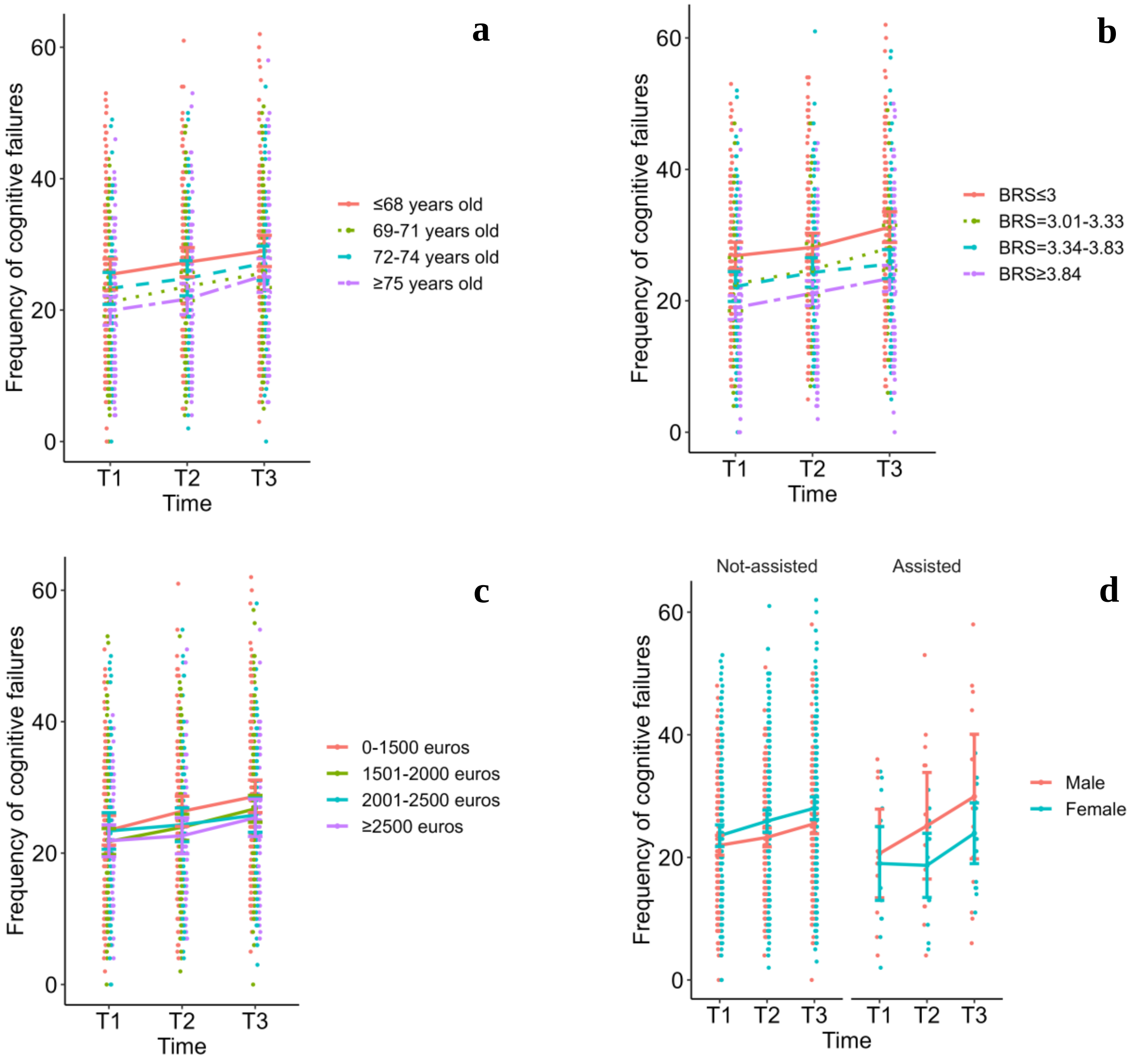

Note. Panel a shows age and changes in frequency of cognitive failures; Panel b shows resilience, measured with BRS (Brief Resilience Scale), and changes in frequency of cognitive failures; Panel c shows monthly net income and changes in frequency of cognitive failures; Panel d shows the interaction between gender and living assisted or not and changes in frequency of cognitive failures.
